# A systematic review and meta‐analysis of the effectiveness of meal replacements for weight loss

**DOI:** 10.1111/obr.12816

**Published:** 2019-01-24

**Authors:** Nerys M. Astbury, Carmen Piernas, Jamie Hartmann‐Boyce, Sophia Lapworth, Paul Aveyard, Susan A. Jebb

**Affiliations:** ^1^ Nuffield Department of Primary Care Health Sciences University of Oxford Oxford UK; ^2^ National Institutes for Health Research (NIHR) Oxford Biomedical Research Centre Oxford UK

**Keywords:** diet, meal replacement, obesity, weight loss

## Abstract

Meal replacements (MR) are generally not recommended in clinical guidelines for the management of obesity. The aim of this review is to provide an up‐to‐date systematic evaluation of the effect of weight loss interventions incorporating MR compared with alternative interventions on weight change at 1 year in adults with overweight or obesity. Six electronic databases were searched from inception to the end of August 2018 for randomized controlled trials comparing the effect of MR with interventions that did not include MR on weight at 1 year. We excluded studies using diets providing <3347 kJ/(800 kcal)/day and those which used total diet replacement (TDR) from this review. Risk of bias was assessed using the Cochrane risk of bias tool. Twenty‐three studies with 7884 adult participants were included. Six out of 23 studies were judged at low risk of bias across all domains, and 5/23 studies were judged at high risk of bias in at least one domain. Studies with similar intervention and comparators were grouped into five comparisons for analysis. Mean weight change at 1 year favoured the MR group relative to the control group in each comparison. In those comparisons where we conducted meta‐analysis, in people assigned to a diet incorporating MR, mean difference was −1.44 kg (−2.48 to −0.39 kg; *I* ^2^ = 38%) compared with alternative kinds of diets. In those assigned to a MR diet along with support, mean difference was −2.22 kg (−3.99 to −0.45, *I* ^2^ = 81%) compared with other diets with support and −3.87 kg (−7.34 to −0.40; *I* ^2^ = 60%) compared with other kinds of diet without support. In those assigned a MR diet with an enhanced level of support, mean difference was −6.13 kg (−7.35 to −4.91, *I* ^2^ = 19%) compared with alternative diets and regular support. Programmes incorporating meal replacements led to greater weight loss at 1 year than comparator weight loss programmes and should be considered as a valid option for management of overweight and obesity in community and health care settings.

**Abbreviations**
BOCF: Baseline observation carried forward
HbA1c: Haemoglobin A1C
HDL cholesterol: High‐density lipoprotein cholesterol
LDL cholesterol: Low‐density lipoprotein cholesterol
MR: Meal replacements
RCT: Randomized controlled trial

## 1 INTRODUCTION

Obesity is a condition of excess accumulation of body fat that causes premature mortality. It also causes substantial morbidity, including significantly increased risks of type 2 diabetes, cardiovascular disease, and several nonsmoking‐related cancers, as well as physical impairments linked to excess weight such as breathlessness, joint problems, and back pain.^1^, ^2^ Nevertheless, the health risks of obesity are offset by weight loss,^3^, ^4^, ^5^, ^6^ with greater weight losses associated with greater health benefits.^7^ The prevalence of excess weight is high and rising throughout the world;^8^ thus, unless effective obesity treatment and prevention strategies are implemented, the increasing incidence of preventable diseases will place a growing burden on health systems and the global economy.^9^ There is a pressing need to identify effective interventions to treat obesity that can be delivered at scale.

Weight loss programmes that incorporate self‐management strategies, such as the use of meal replacements (MR), may be a particularly useful approach, as they can be delivered within the community, potentially without support by health care professionals. For the purposes of this review, we defined MR as discrete foods, food products, or drinks that are used to replace foods usually consumed at one or more meals with the intent to reduce daily energy intake for the purposes of achieving weight loss or weight maintenance following weight loss. MR programmes replace at least one meal per day and include at least one meal comprising conventional foods. This definition not only includes products purposefully marketed as MR for weight loss such as soups, shakes, and bars but also includes portion‐controlled ready meals as well as discreet portions of readily available conventional foods such as breakfast cereal or rice. This review does not include very‐low‐energy diets (VLED) providing less than 3347 kJ (800 kcal)/day, or those that use low‐energy total diet replacement (TDR), which use formula food products to replace all meals and snacks, but the impact of these types of programs on weight has been reviewed by others recently.^10^, ^11^

A previous systematic review and meta‐analysis of interventions that incorporated MR for weight loss, using a similar definition as here, identified only six studies of MR programmes versus comparator. It is reported that the use of MR significantly increased short‐term weight loss (3 months) compared with control. However, at 1 year, there was no statistically significant difference in weight change between the groups, though the point estimate favoured the intervention.^12^ Uncertainty over the long‐term effectiveness of MR may explain why many national guidelines do not reference MR,^13^, ^14^, ^15^, ^16^ or advise against their use^17^ for the routine management of overweight and obesity.

However, in recent years, several further studies of the effects of MR with longer term follow‐up have been conducted. We therefore set out to conduct an up‐to‐date systematic review of randomized controlled trials to examine the effectiveness of MR in people with overweight and obesity, compared with interventions that do not include MR, on weight loss at 1 year. The effects on cardiometabolic risk and any adverse effects were also investigated. We included interventions intended for the treatment of overweight or obesity and considered suitable for use in the community, without medical supervision.

## 2 METHODS

A protocol for this review was published in advance and implemented without changes.^18^

### 2.1 Search strategy

MEDLINE, Embase, PsycINFO, CINAHL, Web of Science, and the Cochrane Controlled Register of Trials (CENTRAL) were searched from database inception to August 2018. We also screened references from systematic reviews identified through our search and requested papers from authors for those we were unable to obtain or for which we had abstracts only. The searches were not restricted by country or language. The search strategy (MEDLINE) is included in Supplementary Table S1.

Studies were included if they recruited adults (≥18 years) with a body mass index (BMI) ≥25 kg/m^2^. Studies in pregnant women, people with eating disorders, those who had undergone bariatric surgery, or in those that included concomitant use of pharmacotherapies for the purposes of weight loss were excluded. We included randomized controlled trials of interventions incorporating the use of one or more MR daily, as part of a hypoenergetic diet intended for weight loss. We excluded interventions in which daily energy intake was restricted to <3347 kJ (800 kcal)/day, as these diets (VLEDs) require medical supervision and have been systematically reviewed previously.^10^ Studies that used formula TDR were also excluded. To be included, studies must have had as a comparator either a weight loss intervention that did not include MR or offered no or minimal intervention. Studies which compared different types of MR but did not include a control arm were excluded. Studies were required to report participants' weight at least 1 year after enrolment.

Covidence (Cochrane) was used for abstract and full‐text screening and data extraction.^19^ Data were extracted on weight and adverse events at all reported time points as well as on fasting glucose; insulin; HbA1c; total, LDL, and HDL cholesterol; triglycerides; and systolic and diastolic blood pressure at 1 year. Studies were excluded if they did not present sufficient information to allow data extraction or quality assessment and if this information was not available in a trial protocol or provided to us on request by the authors. Initially, titles and abstracts were assessed for inclusion by two independent reviewers with disagreements resolved by discussion or by a third reviewer. Full papers of the included studies were obtained and independently assessed for inclusion by two reviewers, with disagreements resolved by discussion or by consultation with a third reviewer.

### 2.2 Data extraction

Two reviewers independently used a prespecified and piloted data extraction form to characterize the population, intervention, control groups, and outcomes and to assess risk of bias. Disagreements were resolved by discussion or by a third reviewer. Authors were contacted for missing data and clarifications as required. Risk of bias was assessed using the Cochrane risk of bias tool,^20^ consistent with the methods used in previous reviews of weight loss interventions.^21^ If the outcome assessors were not blinded, we judged studies at low risk of detection bias where the outcome was objective (eg, body weight measured by study staff). Studies were judged at high risk of attrition bias if fewer than 50% of participants were measured at follow‐up or if there was a difference of ≥20% drop‐out rate between groups, for the primary outcome of weight at 1 year from baseline.

We categorized interventions including MR, and their comparators, to ensure that we compared effectiveness within meaningful groups. There were three types of active intervention. The first was stand‐alone dietary advice which recommended the use of MR to replace one or more eating occasions with the remainder of the diet provided by conventional food (MR diet). The second included interventions which recommended the use of MR and also provided participants with additional support to lose weight (MR diet + support). If the “support” offered was at a higher intensity than the support offered to the comparator group, we called this enhanced support, and this made up the third intervention group (MR diet + enhanced support). There were three types of comparators. The first comprised advice on dietary changes to aid weight loss that did not include the use of MR (diet only). The second was broader support for weight loss, which include advice on diet and other behavioural strategies, though the details may not be specified (diet + support). The third type of comparator intervention offered a nominal intervention for weight loss, either in the form of a printed leaflet or one‐off educational lecture (minimal intervention).

### 2.3 Analysis

Meta‐analyses were conducted in Review Manager 5.2 (Cochrane)^22^ to examine the difference between intervention and comparator groups for weight change at 1 year (primary outcome). Additionally, where available, we extracted interim weight change (~3 months, typically at the end of the main intervention) and weight change at 2 and 4 years after baseline. Further analyses were conducted to examine the changes in biomarkers of diabetes and cardiovascular risk at 1 year. Studies varied in whether and how they imputed data for participants lost to follow‐up, and as the practices adopted for this can impact on the reported effect size, we recalculated weight change using the method of baseline observation carried forward (BOCF) to reduce spurious heterogeneity.^23^

In studies that did not report baseline weight for the randomized participants, we assumed that there was no difference in baseline weight between those randomized and those who completed the study unless it was stated otherwise. In one study, it was unclear how many participants were lost to follow‐up in each group, and we assumed equal drop‐out for the purposes of the BOCF calculation.^24^ A sensitivity analysis was conducted removing this study from the comparison.

Because the definitive numbers of participants achieving ≥5% or ≥10% weight loss were inconsistently reported in the included papers, we estimated these numbers based on the mean and standard deviation of the reported weight loss, assuming a normal distribution as a post hoc analysis.

All meta‐analyses used a random effects model to account for differences in the intervention programmes and populations. Pooled results were calculated as mean differences in kilograms (kg) with 95% confidence intervals (CI) for continuous variables; weighted means were used to report individual group means. For the proportion of participants achieving ≥5% or ≥10% weight loss, pooled results are reported as odds ratio with 95% confidence intervals. The *I* ^2^ statistic was used to quantify statistical heterogeneity.^25^ We used Cochrane guidance for interpretation of *I* ^2^: 0% to 40% might not be important, 30% to 60% may represent moderate heterogeneity, 50% to 90% may represent substantial heterogeneity, and 75% to 100% considerable heterogeneity.^20^ Where a study contributed more than one intervention arm to a meta‐analysis, the control group was divided equally between interventions to avoid double counting in the pooled result.

In the preregistered protocol, we planned to use meta‐regression to investigate whether characteristics of the MR interventions impacted the observed effect. However, there were few studies in each meta‐analysis, and the data reporting the details of the programmes were limited such that we judged that the outcomes would not be robust, and this was omitted from our analysis.

## 3 RESULTS

**Figure 1:**
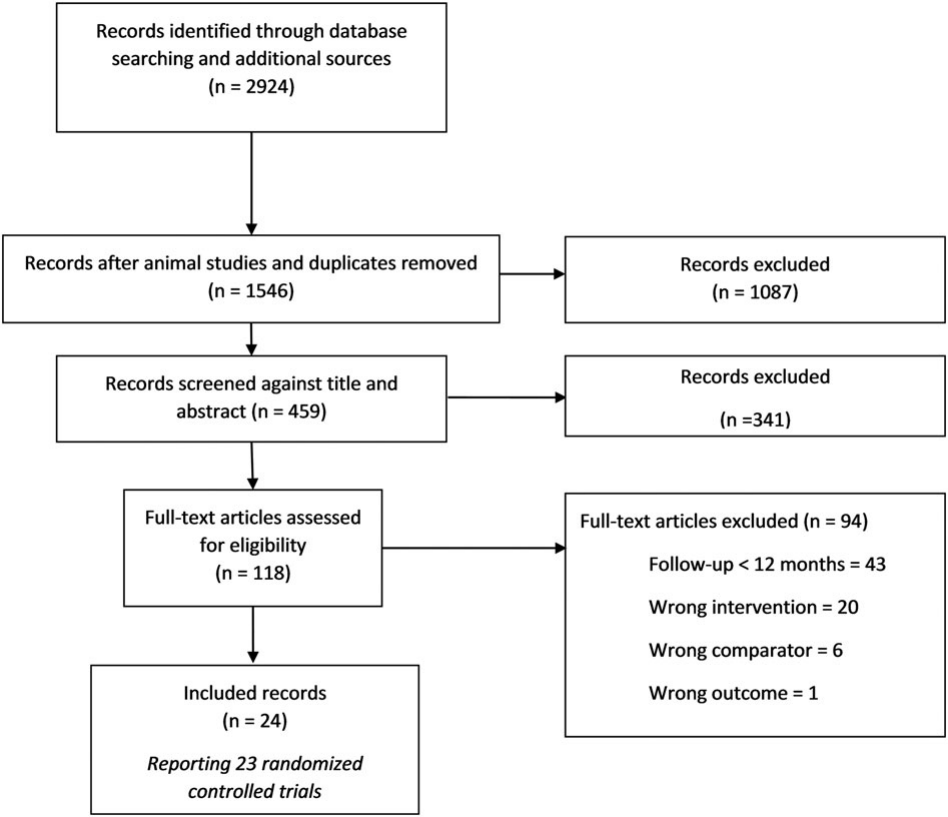
Prisma flow diagram

The literature search identified a total of 2924 papers. One hundred eighteen full papers were retrieved, and 24 papers reporting the results of 23 randomized controlled trials (RCTs) with 8253 participants were eligible for inclusion. The main reason studies were excluded is that they did not report body weight at 1‐year follow‐up or longer, and this information could not be obtained from authors. For some studies, additional data were obtained from the authors.^26^, ^27^, ^28^ The PRISMA flow chart is displayed in Figure 1.

### 3.1 Characteristics of included studies

There were 4411 people allocated to an intervention including MR and 3852 people to a comparator in the included studies. One study was conducted in Australia, 1 in China, 3 in Germany, 1 in Malaysia, 1 in Singapore, 1 in Thailand, and 15 in the United States. Most studies were small; the median number of participants per study was 110 [IQR 92.5 to 165.5]. Over three quarters of participants were women, median 79.0% [IQR 54.2 to 94.2]; the mean participant age was 47.7 years [IQR 41.9 to 52.6], and mean baseline BMI was 34.5 kg/m^2^ [IQR 31.3 to 36.1] (Table 1). There was no significant difference in baseline body weight between intervention and control groups (mean ± SD; http://93.7.kg ± 12.5 and 98.4 ± 12.3 kg in MR and control groups, respectively).

**Table 1:** Characteristics of included studies

Study ID	Location	Funder	Population	Intervention	Comparator	Relevant Outcomes	Follow‐Up Rate
MR diet vs diet only							
Ahrens 2003	USA	SlimFast	N = 95 BMI >25 kg/m^2^ 87% Female	12‐week weight loss: Replace 2 of 3 meals with a liquid MR plus one self‐selected meal of conventional foods 10‐week weight maintenance: 1 MR and 2 self‐selected meals of conventional foods	12‐week weight loss: Self‐selected diet, based on diabetic exchange ~5021 kJ (1200 kcal)/day for women, 6276 kJ (1500 kcal)/day for men 10‐week weight maintenance: Healthy self‐selected diet and control of energy intake as desired	Weight BMI	20% (Not reported by group)
Cheskin 2008	USA	Medifast Inc.	N = 119 BMI 25‐40 kg/m^2^ Type 2 diabetes 56% Female	34‐week weight loss: 25% energy deficit diet with 50%‐60% energy provided by MR suitable for diabetics, the remainder provided by self‐selected conventional foods. 52‐week weight maintenance: Rerandomized to either 26‐week MR followed by 26‐week control intervention, or 26‐week control intervention followed by 26‐week MR.	34‐week weight loss: 25% energy‐deficit diet ADA recommendations 52‐week maintenance: Continued at a lower energy restriction for maintenance	Weight BMI Body fat (%) Waist circumference Fasting glucose Insulin Total cholesterol Systolic blood pressure Diastolic blood pressure Triglycerides HbA1C Medication use	MR: 57% Control: 29%
Ditschuneit 2001	Germany	SlimFast	N = 100 BMI 25‐40 kg/m^2^ 79% Female	3‐month weight loss: 2 MR + regular meal 48‐month weight maintenance: Replace 1 meal and 1 snack with MR.	3‐month weight loss: Low‐energy diet of conventional foods providing 5021 to 6276 kJ (1200 to 1500 kcal)/day. Three meals (breakfast, lunch, and dinner) and two snacks were recommended. 48‐month weight maintenance: Replace 1 meal and 1 snack with MR.	Weight Waist circumference Fasting glucose Insulin Total cholesterol LDL cholesterol Systolic blood pressure Diastolic blood pressure	MR 84% Control: 72%
Khoo 2011	Australia	National Heart Foundation Australia and Medical Benefits Foundation	N = 31 BMI >30 kg/m^2^ WC >102 cm Type 2 diabetes 0% Female	52‐week weight loss: 8 weeks: Energy deficit 2 MR + small meal of self‐selected conventional foods. 44 weeks: Switched to the control diet	52‐week weight loss: High protein ~2510 kJ (600 kcal) energy‐deficit diet Based on the Commonwealth Scientific Industrial and Research Organisation (CSIRO) total well‐being diet	Weight Waist circumference Plasma glucose Insulin Triglycerides LDL cholesterol HDL cholesterol	MR: 47% Control: 58%
Khoo 2013	Singapore	The SingHealth Foundation and Changi General Hospital	N = 48 BMI >27.5 kg/m^2^ Men with erectile dysfunction 0% Female	12‐week weight loss: ~1674 kJ (400 kcal) energy deficit comprising 2 liquid MR + self‐selected meal	12‐week weight loss: Conventional diet ~1674 kJ (400 kcal) energy deficit	Weight BMI Waist circumference Body fat (%) Plasma glucose Plasma insulin Systolic blood pressure Diastolic blood pressure	MR: 58% Control: 54%
Rothacker 2001	USA	Maryland Chapter of the Arthritis Foundation	N = 75 BMI 25‐32 kg/m^2^ 100% Female	1‐year weight loss: Replace 1 or more meals with liquid MR	1‐year weight loss: Low‐energy low‐fat diet of ~5021 kJ (1200 kcal)/day	Weight Fat mass Lean body mass Fat (%)	MR: 84% Control: 87%
	MR diet + support vs diet + support						
Ashley 2001	USA	Slim fast	N = 113 BMI 25‐50 kg/m^2^ Premenopausal women 100% Female	1‐year weight loss: *MR (dietician led):* Small classes (8‐10 participants per class) Weekly for the first 3 months, biweekly for the next 3 months and monthly for the final 6 months. Diet consisting of replacing 2 of the 3 main meals (breakfast, lunch, or dinner) with MR shakes or bars. *MR physician/nurse led:* Brief (10/15 min) biweekly visits with a physician or nurse. Diet consisting of replacing 2 of the 3 main meals (breakfast, lunch, or dinner) with MR shakes or bars.	1‐year weight loss: Small dietician‐led classes (8‐10 participants per class) Weekly for the first 3 months, biweekly for the next 3 months and monthly for the final 6 months. All meals and snacks prepared from self‐selected conventional foods ~5021 kJ (1200 kcal)/day using the US Department of Agriculture food guide pyramid.	Weight Waist circumference Body fat (skinfold) Glucose Insulin Total cholesterol LDL cholesterol HDL cholesterol Triglycerides Systolic blood pressure Diastolic blood pressure	65% overall (not reported by group)
Ashley 2007	USA	SlimFast/Unilever	N = 96 BMI 25‐35 kg/m^2^ 100% Female	1‐year weight loss: Replace 2 of 3 main meals with MR drinks or bars. Bimonthly classes for 6 months followed by monthly classes for 6 months.	1‐year weight loss: Diet plan based of ~5021 kJ (1200 kcal)/day based on self‐selection of conventional foods for meals and snacks using the USDA food guide pyramid. Bimonthly classes of 6 months followed by monthly classes for a further 6 months	Weight (kg) BMI Body fat (%) Waist circumference	MR: 73% Control: 73%
Chaiyasoot 2018	Thailand	Slimwell	N = 110 BMI >25 Metabolic syndrome 83% Female	12 weeks: Replace 2 main meals with one MR for the duration of the 12 week intervention. Group session at baseline followed by 4 individual sessions with dietician.	12 weeks: Baseline group session and 4 individual sessions with dietitian who advised an energy restriction diet based on ~2092‐4184 kJ (500‐100 kcal)/day energy restriction.	Weight (kg)	MR: 72% Control:84%
Chee 2017	Malaysia	Abbott Nutrition Malaysia	N = 230 BMI >23 kg/m^2^ Type 2 diabetes 63% Female	6 months MR (MI): Replace 1 or 2 meals with a diabetes‐specific formula meal replacement. Behavioural support provided using motivational interview. MR (CC): Replace 1 or 2 meals with a diabetes‐specific formula meal replacement. Behavioural support provided using conventional counselling.	6 months: Followed the clinical care pathway of the Malaysian clinical practice guidelines for type 2 diabetes mellitus (2009) and received advice to follow a conventional low‐calorie diet plan 5021 kJ (1200 kcal) or 6276 kJ (1500 kcal)/day using normal foods with standard diabetes support and lifestyle education.	Weight (kg)	MR (MI): 88% MR (CC): 70% Control: 85%
Davis 2010	USA	Medifast Inc.	N = 90 BMI 30‐50 kg/m^2^ 71% Female	16‐week weight loss: 5 MR + a self‐selected meal. Biweekly consultations with a dietician for dietary and behavioural counselling. 24‐week weight maintenance: 2 meetings with dietician at 12‐week intervals.	16‐week weight loss: ~4184 kJ (1000 kcal)/day diet plan plus multivitamin supplement. Biweekly consultations with a dietician for dietary and behavioural counselling. 24‐week weight maintenance: 2 meetings with dietician at 12‐week intervals.	Weight (kg) Waist circumference Body fat (%) Total cholesterol HDL cholesterol LDL cholesterol Triglycerides Systolic BP Diastolic BP	MR: 58% Control: 44%
Flechtner‐Mors 2010	Germany	University of Ulm and Herbalife Inc.	N = 100 Overweight and obese (BMI 27‐45 kg/m^2^), metabolic syndrome 80% Female	3‐month weight loss: Diet instructions to follow diet high protein with energy deficit of ~2092 kJ (500 kcal)/day based on consuming 2 protein‐enriched MR plus one conventional meal and 2 snacks. 9‐month weight maintenance: 1 MR two conventional meals and two snacks	3‐month weight loss: Diet instructions to follow diet with conventional protein content with energy deficit of ~2092 kJ (500 kcal)/day, 3 conventional meals 2 snacks 9‐month weight maintenance: 2 conventional meals 2 conventional snacks and 1 MR	Weight BMI Body fat (%) Blood glucose Insulin HbA1c Total cholesterol HDL cholesterol Triglycerides Adverse events	MR: 56% Control: 78%
Li 2005	USA	Not disclosed‐meal replacements provided by SlimFast foods Co. Inc.	N = 104 BMI 27‐40 kg/m^2^ Type 2 diabetics 62% Female	1 year: Individual consultation with a dietician at baseline and weekly for 2 months, then monthly for the remainder *First 5 days*: 3 soy MR and advice to add fruits and vegetables to their intake *Day 6‐3 months*: 2 MR plus one meal of self‐selected foods *3 months‐1 year:* 1 or 2 MR plus 2 sensible meals.	1 year: Individual consultation with a dietician at baseline and weekly for 2 months, then monthly for the remainder. Individualized meal plan based on ADA exchanges, target reduction in intake of ~2092 kJ (500 kcal)/day.	Weight BMI Glucose Insulin HbA1c Total cholesterol LDL cholesterol HDL cholesterol Triglycerides	MR: 81% Control: 67%
Lowe 2018	USA	National Institutes of Health (NIH)	N = 181 BMI 27‐45 kg/m^2^ 84% Female	1 year: Weekly groups for 6 mo, then biweekly for 6 mo. During months 1‐6 participants were advised to replace 2 meals with MR product. In months 7‐12 switched to replacing 1 meal and 1 snack with MR product.	1 year: Weekly groups for 6 mo, then biweekly for 6 mo. Following materials from lifestyle, exercise, attitude relationships and nutrition (LEARN) and diabetes prevention program manual. Focus on weight loss months 1‐6 and weight maintenance months 7‐12.	Weight (kg)	MR: 73% Control: 81%
Ptomey 2017	USA	National Institutes of Diabetes, Digestive and Kidney Diseases (NIDDK)	N = 150 BMI >25 kg/m^2^ Intellectual developmental disabilities 57% Female	18‐month weight loss: Participants and study partners taught how to prepare and consume 2 portion‐controlled main meal MR and 2 MR shakes a day with additional meal of self‐selected conventional foods according to a guide. Health educators conducted monthly home visits.	18‐month weight loss: Participants and study partners taught how to follow a 2092‐2929 kJ (500‐700 kcal)/day energy deficit diet following Myplate approach. Health educators conducted monthly home visits.	Weight BMI Waist circumference	MR: 69% Control: 65%
Rolls 2017	USA	National Institutes of Diabetes, Digestive and Kidney Diseases (NIDDK)	N = 124 BMI 28‐45 kg/m^2^ 100% Female	1‐year weight loss: Participants met individually with a dietician for 19 sessions. They were instructed to eat preportioned main dishes daily for lunch and dinner during months 1‐3 of the study and were encouraged to continue this practice subsequently.	1‐year weight loss: Participants met individually with a dietician for 19 sessions. Instructed to follow US dietary guidelines emphasizing eating less whilst making healthy choices from all food groups.	Weight Glucose HbA1c Total cholesterol LDL cholesterol HDL cholesterol Triglycerides Systolic blood pressure Diastolic blood pressure	MR: 82% Control:82%
MR diet + support vs diet only							
Rock 2007	USA	Jenny Craig Inc.	N = 70 BMI 25‐40 kg/m^2^ Minimum 15 kg overweight 100% F	12‐month weight loss: Weekly one‐to‐one contacts with a counsellor, with follow‐up telephone and e‐mail contacts and web site/message board availability. Energy reduced diet with prepackaged prepared foods typically providing 3347 kJ (800 kcal)/day, with the remainder provided by self‐selected conventional food. A goal is 30 minutes of physical activity on 5 or more days of the week.	1‐year weight loss: Consultation with a dietician at baseline and 16 weeks who provided a diet and physical activity plan to promote weight loss.	Weight BMI Waist circumference Hip circumference Insulin Total cholesterol LDL cholesterol HDL cholesterol Triglycerides	MR: 91% Control: 94%
Shikany 2013	USA	Medifast Inc.	N = 120 BMI 30‐50 kg/m^2^	26‐week weight loss: Quick start guide for dietary plan 5 MR (3347‐4184 kJ (800‐1000 kcal)/day) plus 1 self‐selected meal of conventional food. Online access to resources, dietician trainers, message boards, chat rooms. 26‐week weight maintenance: Energy intake to achieve weight maintenance with the option to include 0‐3 MR a day.	26‐week weight loss: 4184 kJ (1000 kcal)/day mean plan based on self‐selected, self‐prepared conventional foods. Referral to the http://MyPyramid.gov website for nutritional information. Advised to take multivitamin supplement. 26‐week weight maintenance: Energy intake to achieve weight maintenance based on self‐selected conventional foods	Weight BMI Waist circumference Fat mass Fat‐free mass Systolic BP Glucose Total cholesterol LDL cholesterol HDL cholesterol Triglycerides Diastolic BP	MR: 83% Control: 75%
MR diet + enhanced support vs diet + support							
Rock 2010	USA	Jenny Craig Inc.	N = 442 BMI >25 kg/m^2^ Minimum 15 kg overweight 100% Female	2‐year weight loss: Weekly contacts for up to 2 years. Meal plan consisting of low‐fat reduced energy diet, with prepackaged foods providing up to 70% energy requirements, with remainder from self‐selected conventional foods. Group was split into 2 groups: Telephone advice: Counselling provided over the telephone or via email In‐person advice: Counselling provided face‐to face	2‐year weight loss: Consultation with a research dietetic professional at baseline and 6 months. Provided with print material describing dietary and physical activity guidelines to promote weight loss.	Weight BMI Waist circumference Total cholesterol LDL cholesterol HDL cholesterol Triglycerides	MR (in person): 99% MR (telephone): 100% Control: 98%
Rock 2014	USA	Jenny Craig Inc.	N = 70 BMI 25‐40 kg/m^2^ Minimum 15 kg overweight 100% F	9‐month weight loss: *Months 1‐6:* Three main meals and one to two snack MR provided for 7 days/week *Months 7‐9:* Three main meals and one to two snack MR provided for And for 5 days/week 3‐month weight maintenance: One main meal and one snack MR provided each day for use as desired. Weekly consultations with train counsellors were recommended during the first 9 months after which participants had the option to move to biweekly or monthly consultations.	1‐year weight loss: Consultation at baseline and at 6 months with a research dietician who provided print materials on dietary and physical activity guidelines for weight loss and weight loss maintenance	Weight BMI Waist circumference Total cholesterol LDL cholesterol HDL cholesterol Triglycerides Insulin	MR: 91% Control: 94%
MR diet + support vs minimal intervention							
Kempf 2017	Germany	Almased	N = 409 BMI ≥27 kg/m^2^ Poorly controlled type 2 diabetes (HbA1c ≥ 7.5%) 42% Female	52 weeks: MR (stringent use of MR): *Week 1:* Replace 3 meals/day with MR and include 45 g oil rich omega 3 fatty acids and 750 mL vegetable juice. *Weeks 2‐4:* Replace 2 meals/day with MR and low‐carb other meals *Weeks 5‐52*: Replace 1 meal/day with MR MR (moderate use of MR): *Week 1*: Replace 2 meals/day with MR and include 45 g oil rich omega 3 fatty acids and 750 mL vegetable juice. *Weeks 2‐4:* Replace 2 meals/day with MR and low‐carb other meals *Weeks 5‐52:* Replace 1 meal/day with MR	Usual care: Quarterly visits with physician for routine care as defined by the disease management programs (DMP) for type 2 diabetes in Germany.	Weight (kg) Blood glucose Total cholesterol LDL cholesterol HDL cholesterol Triglycerides Systolic blood pressure Diastolic blood pressure	MR (stringent):69% MR (moderate):70% Control:74%
Look Ahead Research Group 2007	USA	NIH	N = 5145 BMI >25 kg/m^2^ (BMI >27 kg/m^2^ if taking insulin) Type 2 diabetic 60% F	1‐year weight loss: *Months 1‐6:* weekly session plus 3 group sessions per month *Months 7‐12:* biweekly group sessions plus one individual session. All sessions comprised of advice and support from dieticians, registered psychologists and exercise specialists to encourage >10% weight loss from initial body weight.	Diabetes support and education: Diabetes education session and invited to attend 3 additional group sessions during the 1^st^ year.	Weight BMI Waist circumference HbA1c LDL cholesterol HDL cholesterol Triglycerides Systolic blood pressure Diastolic blood pressure	MR: 97% Control: 96%
Xu 2013	China	The Science and Technology Commission of Shanghai Municipality (07ZR14036) and the Public Health Bureau of Shanghai (2007168)	N = 88 BMI >18.5 kg/m^2^ Impaired glucose regulation 52% F	12‐week weight loss: Educational lecture on balanced diet, regular exercise, and behavioural strategies. Encouraged to follow the “2007 Chinese guidelines for the management of type 2 diabetes” and “Dietary Guidelines for Chinese” Weekly intensive lifestyle meetings and weekly medical evaluation by a physician.	Educational lecture: Educational lecture on balanced diet, regular exercise, and behavioural strategies. Encouraged to follow the “2007 Chinese guidelines for the management of type 2 diabetes” and “Dietary Guidelines for Chinese”	Weight BMI Waist circumference Total cholesterol HDL cholesterol LDL cholesterol Triglycerides Fasting plasma glucose HbA1c Systolic blood pressure Diastolic blood pressure	MR: 89% Control: 95%

Fourteen studies were conducted in people who were overweight but with no specific clinical condition,^24^, ^27^, ^28^, ^29^, ^30^, ^31^, ^32^, ^33^, ^34^, ^35^, ^36^, ^37^, ^38^, ^39^ five in people with type 2 diabetes,^40^, ^41^, ^42^, ^43^, ^44^ two in participants with metabolic syndrome or impaired glucose regulation,^45^, ^46^ one in men with type 2 diabetes and erectile dysfunction,^47^ and one in adults with developmental disabilities and obesity.^48^

From the 23 studies reporting weight change data at 1 year (Figure 2), 17 of these studies reported weight change at a point earlier than 1 year (Figure 3), which was collected at a median 18 weeks (range 8 to 34 weeks). Six studies also reported weight outcomes at 2 years (range 64 to 108 weeks) (Figure 4), and two studies reported weight outcomes at 4 years (range 204‐208 weeks) (Figure 5). Twelve studies reported information on biomarkers of cardiovascular and diabetes risk at 1 year (Table 3 and Figures S1‐S9).

**Figure 2:**
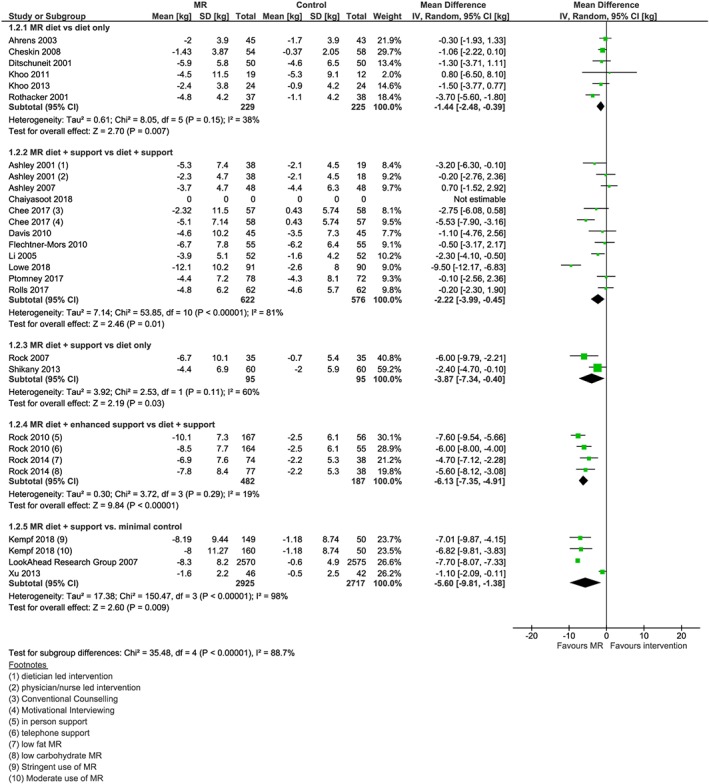
Forest plot of mean weight change (kg) from baseline at 1 year between interventions incorporating meal replacements (MR) and control interventions by comparison group [Colour figure can be viewed at http://wileyonlinelibrary.com]

**Figure 3:**
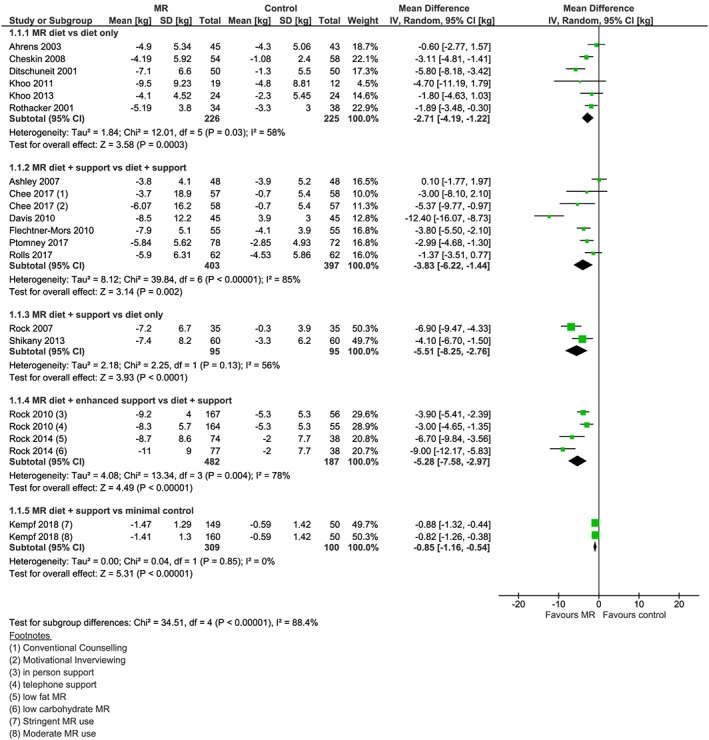
Forest plot of interim (>1 year) mean weight change (kg) from baseline between interventions incorporating meal replacements (MR) and control interventions by comparison group [Colour figure can be viewed at http://wileyonlinelibrary.com]

**Figure 4:**
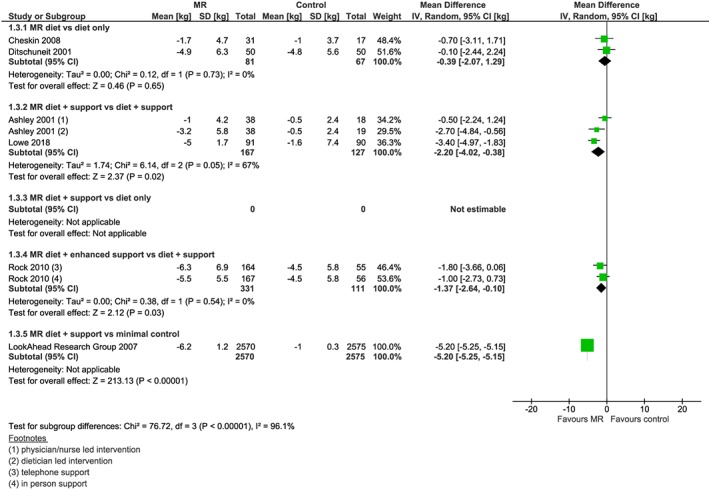
Forest plot of mean weight change (kg) from baseline at 2 years between interventions incorporating meal replacements (MR) and control interventions by comparison group [Colour figure can be viewed at http://wileyonlinelibrary.com]

**Figure 5:**
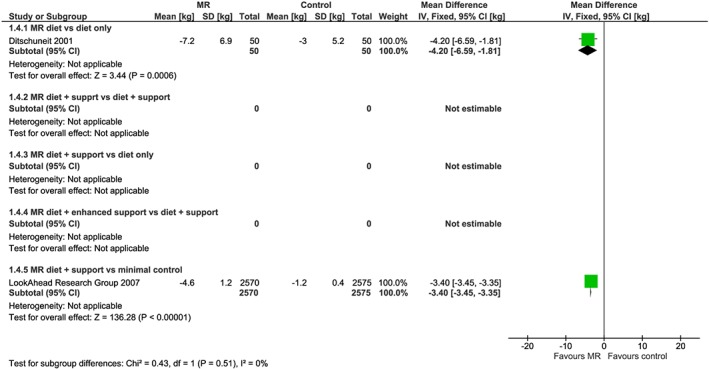
Forest plot of mean weight change (kg) from baseline at 4 years between interventions incorporating meal replacements (MR) and control interventions by comparison group [Colour figure can be viewed at http://wileyonlinelibrary.com]

### 3.2 Interventions and comparators

In 15 studies (65%), the MR used consisted of specially formulated shakes, soups, or bars.^24^, ^27^, ^29^, ^30^, ^32^, ^33^, ^34^, ^37^, ^38^, ^39^, ^41^, ^43^, ^44^, ^45^, ^47^ In four studies (17%), the MR consisted of prepackaged portion‐controlled meals which participants reconstituted or cooked at home,^28^, ^35^, ^36^, ^42^ and in four studies (17%), the MR was a combination of prepackaged portion‐controlled meals and specially formulated shakes, soups, or bars.^31^, ^38^, ^40^, ^48^

The median duration of the active weight loss intervention in all 23 included studies was 20 weeks [IQR 12 to 52 weeks]. Twelve studies also included a second MR phase for continued weight loss or weight maintenance where continued use of MR was recommended, generally at a reduced level. The median duration of this phase was 42 weeks [IQR 30 to 52 weeks].^28^, ^30^, ^31^, ^32^, ^33^, ^34^, ^38^, ^42^, ^44^, ^46^, ^47^, ^48^

During the weight loss phase, 13 studies advised participants to replace two eating occasions each day with a MR.^24^, ^27^, ^28^, ^29^, ^30^, ^32^, ^33^, ^34^, ^41^, ^43^, ^44^, ^45^, ^47^ One study advised participants to replace one eating occasion,^39^ and one, two, and four studies advised participants to replace three,^37^ four,^26^, ^48^ or five^31^, ^38^, ^40^, ^42^ eating occasions (meals or snacks) with one MR, respectively. Two studies (four intervention arms) did not specify the number of MR products participants were advised to consume, but rather recommended that participants replace a proportion of their daily energy intake with the MR products.^35^, ^36^

In six of the included studies, the active intervention consisted of advice to use and/or provision of MR products along with dietary guidance on the quality and quantity of food to be consumed in the remainder of the diet (MR diet).^4^, ^12^, ^19^, ^33^, ^47^, ^49^ In 15 studies, in addition to the MR diet, participants also received support for weight loss during the active intervention. This support consisted of 12 sessions [IQR 7 to 19] over 24 weeks, ranging from 12 to 52 weeks [IQR 12 to 52 weeks] (MR diet + support).^27^, ^28^, ^29^, ^30^, ^31^, ^34^, ^36^, ^38^, ^39^, ^41^, ^43^, ^44^, ^45^, ^46^, ^48^ In two studies (four intervention arms), an enhanced level of support was offered to the MR group (73 sessions [IQR 57.5 to 88.5 sessions] over 78 weeks [IQR 65 to 91 weeks]) compared with the support provided to the control group which consisted of two sessions [IQR 2 to 2] over 78 weeks [IQR 65 to 91 weeks]) (MR diet + enhanced support).^35^, ^42^

In eight studies, the control intervention was dietary advice or a diet plan providing guidance on what to eat to achieve weight loss (diet only).^24^, ^32^, ^33^, ^36^, ^37^, ^38^, ^40^, ^47^ In a further 12 studies, the control diet was combined with support for weight loss, consisting of 11.5 sessions [IQR 4.75 to 18.25] over 52 weeks ranging from 12 to 52 weeks [IQR 45 to 52 weeks] (diet + support).^26^, ^27^, ^28^, ^29^, ^30^, ^31^, ^34^, ^35^, ^42^, ^43^, ^45^, ^48^ In three studies, the control intervention consisted of an information leaflet, educational lecture, or usual care (minimal control).^39^, ^41^, ^44^ Accordingly, the included studies fell into one of five distinct comparisons for analysis:

- MR diet versus diet only^24^, ^32^, ^33^, ^37^, ^40^, ^47^
- MR diet + support versus diet + support^27^, ^28^, ^29^, ^30^, ^31^, ^34^, ^43^, ^45^, ^46^, ^48^
- MR diet + support versus diet only^36^, ^38^
- MR diet + enhanced support versus diet + support^35^, ^42^
- MR diet + support versus minimal intervention^39^, ^41^, ^44^

A full explanation and examples of the interventions that make up the comparison groups are provided in Table S2. Details of the individual interventions in the included studies by comparison group are presented in Table 1.

### 3.3 Risk of bias

Summary risk of bias for the included studies is provided in Table 2, and justifications for any unclear of high judgments are provided in the supplementary material (Table S3). Six of the included studies were judged to be at low risk of bias across all domains.^33^, ^38^, ^41^, ^44^, ^45^, ^48^ Five studies were judged to be at high risk of bias in at least one domain,^24^, ^26^, ^31^, ^34^, ^40^ with all other studies judged to be at unclear risk of bias in at least one domain.^27^, ^28^, ^29^, ^30^, ^32^, ^34^, ^35^, ^36^, ^37^, ^39^, ^40^, ^42^, ^47^ Twelve studies were judged to be at low risk of sequence generation bias,^28^, ^32^, ^33^, ^34^, ^38^, ^40^, ^41^, ^42^, ^43^, ^44^, ^45^, ^48^ with the remaining studies at unclear risk,^24^, ^26^, ^27^, ^29^, ^30^, ^31^, ^35^, ^36^, ^37^, ^39^, ^47^ mainly due to insufficiently detailed reporting. Six studies were judged to be at low risk of allocation concealment bias,^33^, ^38^, ^41^, ^44^, ^45^, ^48^ with the other 17 studies judged to be at unclear risk of this bias because they did not report whether allocation was concealed or not. Five studies were judged to be at high risk of attrition bias,^24^, ^31^, ^34^, ^40^, ^46^ mainly because of unequal follow‐up rates between groups, with all other studies judged to be at low risk of attrition bias (Table 3).^27^, ^28^, ^29^, ^30^, ^32^, ^33^, ^35^, ^36^, ^37^, ^38^, ^39^, ^41^, ^42^, ^43^, ^44^, ^45^, ^47^, ^48^

**Table 2:** Risk of bias for included studies

	Sequence Generation	Allocation Concealment	Blinding of Participants and Personnel	Incomplete Outcome Data	Other Sources of Bias
MR diet vs. diet‐only					
Ahrens 2003	Unclear	Unclear	Low	High	Low
Cheskin 2008	Low	Unclear	Low	High	Low
Ditschuneit 2001	Low	Unclear	Low	Low	Low
Khoo 2011	Unclear	Unclear	Low	Low	Low
Khoo 2013	Low	Low	Low	Low	Low
Rothacker 2001	Unclear	Unclear	Low	Low	Low
MR diet + support vs diet + support					
Ashley 2001	Unclear	Unclear	Low	Low	Low
Ashley 2007	Unclear	Unclear	Low	Low	Low
Chaiasoot 2017	Low	Low	Low	Low	Low
Chee 2018	Low	Unclear	Low	Low	Low
Davis 2010	Unclear	Unclear	Low	High	Low
Flechtner‐Mors 2010	Unclear	Unclear	Low	High	Low
Li 2005	Low	Unclear	Low	High	Low
Lowe 2018	Unclear	Unclear	Low	Low	Low
Ptomey 2017	Low	Low	Low	Low	Low
Rolls 2017	Low	Unclear	Low	Low	Low
MR diet + support vs diet only					
Rock 2007	Unclear	Unclear	Low	Low	Low
Shikany 2013	Low	Low	Low	Low	Low
MR diet + enhanced support vs diet + support					
Rock 2010	Unclear	Unclear	Low	Low	Low
Rock 2014	Low	Unclear	Low	Low	Low
MR diet + support vs minimal control					
Kempf 2017	Low	Low	Low	Low	Low
Look Ahead 2007	Low	Low	Low	Low	Low
Xu 2013	Unclear	Unclear	Low	Low	Low

**Table 3:** Biomarkers of cardiovascular disease risk and glycaemic control

	Studies n	Participants n	Mean Difference	95% CI	*I* ^2^
Fasting blood glucose, mmol/L					
MR diet vs diet only	4	291	−0.14	−0.57, 0.29	79
MR diet + support vs diet + support	3	336	−0.03	−0.24, 0.19	29
MR diet + support vs diet only	1	120	−0.07	−0.2, 0.06	‐
MR diet + enhanced support vs diet + support	1	227	−0.96*	−1.84, −0.07	73
MR diet + support vs minimal intervention	3	5822	−0.60*	−0.85, −0.35	98
Fasting insulin, pmol/L					
MR diet vs diet only	4	291	−12.37*	−17.93, −6.82	94
MR diet + support vs diet + support	3	336	−6.9*	−7.74, −6.06	91
MR diet + support vs diet only	1	70	−36.8*	−53.05, −20.55	‐
MR diet + enhanced support vs diet + support	1	227	1.25	−40.32, 42.83	0
MR diet + support vs minimal intervention	‐	‐	‐	‐	‐
HbA1c, %^a^					
MR diet vs diet only	1	112	−0.8*	−1.19, −0.41	‐
MR diet + support vs diet + support	2	214	−0.18*	−0.22, −0.14	90
MR diet + support vs diet only	‐	‐	‐	‐	‐
MR diet + enhanced support vs diet + support	1	227	−0.55*	−0.74, −0.36	‐
MR diet + support vs minimal intervention	3	5822	−0.49*	−0.52, −0.46	81
Total cholesterol, mmol/L					
MR diet vs diet only	2	212	−0.11*	−0.18, −0.03	0
MR diet + support vs diet + support	5	541	−0.07*	−0.13, −0.01	23
MR diet + support vs diet only	2	190	−0.05	−0.18, 0.08	0
MR diet + enhanced support vs diet + support	2	669	0.04	−0.03,0.12	0
MR diet + support vs minimal intervention	2	499	−0.28	−0.52, 0.05	91
LDL cholesterol, mmol/L					
MR diet vs diet only	2	143	0.11	−0.37, 0.59	77
MR diet + support vs diet + support	4	429	−0.14	−0.29, 0.01	88
MR diet + support vs diet only	2	190	−0.08	−0.28, 0.12	62
MR diet + enhanced support vs diet + support	2	667	0.07	−0.08, 0.21	67
MR diet + support vs minimal intervention	3	5642	−0.12	−0.45, 0.20	99
HDL Cholesterol, mmol/L					
MR diet vs diet only	3	243	0.00	−0.03, 0.03	0
MR diet + support vs diet + support	4	426	−0.01	−0.07, 0.05	87
MR diet + support vs diet only	2	190	0.11	−0.06, 0.28	89
MR diet + enhanced support vs diet + support	2	667	0.06	0.00, 0.11	62
MR diet + support vs minimal intervention	3	5822	0.06*	0.05, 0.07	11
Triglycerides, mmol/L					
MR diet vs diet only	3	243	−0.14	−0.49, 0.21	75
MR diet + support vs diet + support	3	316	0.02	−0.10, 0.15	77
MR diet + support vs diet only	2	190	−0.09	−0.17, 0.00	0
MR diet + enhanced support vs diet + support	2	669	−0.22*	−0.43, −0.02	90
MR diet + support vs minimal intervention	2	5233	0.00	−0.28, 0.29	99
Systolic blood pressure, mmHg					
MR diet vs diet only	2	212	−8.30*	−11.83, −4.76	23
MR diet + support vs diet + support	4	435	−0.33*	−0.6, −0.06	0
MR diet + support vs diet only	1	120	−1.60	−3.86, 0.66	‐
MR diet + enhanced support vs diet + support	1	227	0.34	0.15, 0.53	‐
MR diet + support vs minimal intervention	3	5642	−3.76*	−4.18, −3.34	0
Diastolic blood pressure, mmHg					
MR diet vs diet only	3	260	−2.81*	−5.01, −0.60	55
MR diet + support vs diet + support	3	325	−1.23*	−2.42, −0.04	20
MR diet + support vs diet only	1	120	0.07	−1.20, 1.34	‐
MR diet + enhanced support vs diet + support	1	227	−0.58	−3.28, 2.12	69
MR diet + support vs minimal intervention	3	5642	−1.22*	−1.53, −0.90	11

a HbA1C % units used cannot convert to mmol/mol.

* Statistically significant *P* < 0.05.

### 3.4 Weight change

Twenty‐three studies were included in the primary analysis (weight change at 1 year) across the five comparisons, defined based on intervention and comparator type (Figure 2). Where reported, the difference in weight change reported earlier than 1 year consistently favoured the MR treatment (Figure 3). Results for 1 year or longer follow‐up are reported narratively below. Odds ratios of achieving weight change ≥5% and ≥10% baseline weight at 1 year were calculated for each comparison (Table 4).

**Table 4:** Estimated odds of achieving weight change ≥5% and ≥10% lower than baseline weight at 1 year in MR group compared with control group by comparison

	≥5% Weight Loss	≥10% Weight Loss
	OR [95% CI]	OR [95% CI]
MR diet vs diet only	2.83* [1.37, 5.86] *I* ^2^ = 40	1.73 [0.92, 3.26] *I* ^2^ = 0
MR diet + support vs diet + support	1.49* [1.08, 2.06] *I* ^2^ = 44	1.80* [1.12, 2.87] *I* ^2^ = 56
MR diet + support vs diet only	2.83* [1.37, 5.86] *I* ^2^ = 25	5.95* [2.12, 16.67] *I* ^2^ = 1
MR diet + enhanced support vs diet + support	4.32* [3.01, 6.20] *I* ^2^ = 0	6.63* [4.01, 10.94] *I* ^2^ = 0
MR diet + support vs minimal control	4.03* [1.87, 8.69] *I* ^2^ = 82	8.32* [2.02, 34.16] *I* ^2^ = 93

* Statistically significant *P* < 0.05.

#### 3.4.1 MR diet vs diet only

Six studies compared a MR diet with diet only.^24^, ^32^, ^33^, ^37^, ^40^, ^47^ Mean weight change among participants randomized to an intervention incorporating the use of MR (n 229) was −3.0 kg at 1 year compared with participants in the diet only intervention (n 225) who had weight change of −1.5 kg (mean difference = −1.44 kg; 95% CI −2.48, −0.39, *I* ^2^ = 38%)^24^, ^32^, ^33^, ^37^, ^40^, ^47^ (Figure 2).

Two of the studies in this comparison also reported weight change at 2 years^32^, ^40^ where mean weight change in participants randomized to the MR diet intervention (n 81) was −3.4 kg while those randomized to the diet only intervention (n 67) lost −3.0 kg (mean difference = −0.39 kg; 95% CI −2.07, 1.29, *I* ^2^ = 0%) (Figure 4). One of these studies^32^ reported that 4 years after randomization, mean weight change in those allocated to the MR diet intervention (n 50) was −7.2 kg, while in the comparator group (n 50), it was −3.0 kg (mean difference = −4.2 kg; 95% CI −6.59, −1.81), reflecting continued weight loss in the MR group but little change in weight since 1 year in the comparator group (Figure 5).

#### 3.4.2 MR diet + support vs diet + support

Ten studies (12 intervention arms and 10 control arms) were included in the MR diet + support versus diet + support comparison.^27^, ^28^, ^29^, ^30^, ^31^, ^34^, ^43^, ^45^, ^46^, ^48^ One study was omitted from the meta‐analysis as weight data were not normally distributed, were reported in the paper as median and interquartile ranges, and we could not obtain further data.^45^ In the nine studies included in the meta‐analysis, mean weight change among participants randomized to a MR diet plus support programme (n 622) was −2.4 kg at 1 year, compared with −1.34 kg in a comparator group (n 576) with a diet but no MR and a similar support programme (mean difference = −2.22 kg; 95% CI −3.99 to −0.45, *I* ^2^ = 81%) (Figure 2). Statistical heterogeneity was high, but attributable to one small outlier,^27^ which showed greater weight loss favouring the intervention. In the study omitted from the meta‐analysis, weight change was consistent with the other included studies, in that the median weight change was greater in the MR group (median −1.8 kg, IQR −4.13, 0.43) compared with the control group (median −0.8 kg, IQR −4.0, 1.05).^45^ Two of the included studies also reported weight at 2 years,^27^, ^30^ with weight change favouring MR over control interventions (mean difference −2.2 kg, 95% CI −4.02 to −0.38, *I* ^2^ = 67%) (Figure 4).

#### 3.4.3 MR diet + support vs diet only

Two studies compared interventions involving a MR diet and support programme (n 95) with a diet‐only comparator (n 95).^36^, ^38^ Mean weight change at 1 year was −5.3 kg for the MR diet + support and −1.5 kg in the diet only group (mean difference = −3.87 kg; 95% CI −7.34 to −0.40, *I* ^2^ = 60%) (Figure 2). Neither of these studies reported weight beyond 1 year.

#### 3.4.4 MR diet + enhanced support vs diet + support

Two studies tested a MR diet together with an enhanced support programme.^35^, ^42^ At 1 year, mean weight change among participants randomized to MR diet plus enhanced support (n 482) was −8.5 kg while among those receiving the diet + support (n 187), it was −2.4 kg (mean difference = −6.13 kg; 95% CI −7.35, to −4.91, *I* ^2^ = 19%) (Figure 2). One study (two intervention arms) followed up participants at 2 years,^35^ with weight change (−5.5 kg MR with in person support, −6.3 kg MR with telephone support, and −4.5 kg in control group) continuing to favour the MR diet (n 331) over the comparator (n 111) (mean difference = −1.37 kg; 95% CI −2.64 to −0.10) (Figure 4).

#### 3.4.5 MR diet + support vs minimal intervention

Three studies (four intervention arms) compared a MR diet plus support with a minimal intervention control.^39^, ^41^, ^44^ Statistical heterogeneity was considerable (*I* ^2^ = 98%); therefore, we do not present pooled results here. In all three studies, participants in the intervention arm lost significantly more weight than those in the control arm at 1 year. In a study in China (n = 88), the mean difference was −1.10 kg (95% CI −2.09 to −0.11), favouring the MR group. In a study in Germany (n = 409), mean difference in weight change from baseline was −7.01 kg (95% CI −9.87, to −4.15) in the moderate use of MR group and −6.82 kg (95%CI −9.81 to −3.83) in the stringent use of MR groups compared with control group, respectively.^44^ In the Look Ahead study in the USA (n = 5145), mean difference in weight change between participants in the MR diet + support group compared with the control (usual care) group was −7.70 kg (95% CI −8.07 to −7.33) (Figure 2). In the latter study, the mean difference in weight change was also significantly greater for MR diet plus support group at 2 years (mean difference = −5.20 kg, 95% CI −5.25 to −5.15) and 4 years (mean difference −3.4 kg, 95% CI −3.45 to −3.35) (Figures 4 and 5). The other two studies did not report weight change beyond a year.

### 3.5 Participants achieving >5% and >10% weight loss at 1 year

For each comparison, we calculated the odds ratio that participants would achieve a weight loss ≥5% and ≥10% from baseline at 1 year in the MR group compared with the control (Table 4).

For weight change of ≥5% baseline weight, all comparisons showed that the odds of achieving this were statistically significantly greater in the MR group compared with control. For weight change ≥10%, four out of the five showed that participants allocated to MR intervention had statistically greater odds of achieving the cut‐point compared with those assigned to the control.

### 3.6 Biochemical outcomes

There were very limited data on biochemical outcomes with only 12 studies reporting at least one biochemical outcome.^28^, ^32^, ^33^, ^34^, ^38^, ^39^, ^40^, ^41^, ^42^, ^44^, ^46^, ^47^ Across all studies that reported outcomes, results consistently favoured meal replacement groups for HbA1_C_. For the results for all other biochemical outcomes (glucose, insulin, lipids, and blood pressure), results were mixed and rarely reached statistical significance. Further detail can be found in Table 3 and Figures S1 to S9.

### 3.7 Adverse events

Only two studies reported information on adverse events (AE).^26^, ^41^ Neither of these studies gave details on the total number of AEs reported by intervention arm, but the Look Ahead Research Group 2007 reported that there were no between‐group differences in the frequency of hypoglycaemia, fractures, amputations, congestive heart failure, or occurrence of gallstones among 5145 participants.^50^ Flechtner‐Mors et al reported that there were no adverse events that could be attributed to the intervention.^26^ We also considered nutritional deficiencies to be an AE. Five studies assessed diet quality during the interventions, and these studies reported that diet quality was improved in those randomized to MR.^29^, ^30^, ^32^, ^46^, ^48^

## 4 DISCUSSION

The use of discrete foods, food products, or drinks a replacement for usual foods on some occasions during the day leads to significantly greater weight loss at 1 year than comparator interventions based wholly on conventional foods, with commensurate improvements in HbA1c and mixed findings for markers of diabetes and cardiovascular disease risk. All of the included studies individually reported that interventions including MR products were either equally as effective or superior to the comparator interventions for interim weight change as well as weight change at 1 year. Only two studies reported outcomes at 4 years, but both reported that weight change from baseline to 4 years favoured the MR groups over control. Only two studies assessed adverse events; there was no evidence from these studies of any excess adverse events associated with the use of MR products.^26^, ^41^

A strength of this review is the rigorous methodology, including use of a consistent approach to address missing data (BOCF).^23^ Given the scope of this review, the included studies are inherently clinically diverse. We tried to overcome this by grouping studies according to a predefined protocol to offer a meaningful insight into some of the variation in weight losses seen in studies using different treatment or comparator protocols. However, there is still moderate to substantial statistical heterogeneity in some of the groups. It is plausible that the effect of MR on weight could be affected by the factors that vary between studies, most obviously variations between interventions (nature, frequency, duration, or composition of the MR used) or differences in participant characteristics. However, there were insufficient studies in each comparison to fully explore study characteristics which may explain the observed heterogeneity between studies through meta‐regression or subgroup analyses.

For each domain of bias, the majority of studies were judged at low or unclear risk of bias. Five studies were judged at high risk of attrition bias.^24^, ^31^, ^34^, ^40^, ^46^ These studies were judged at high risk of bias because fewer than 50% of participants were measured at follow‐up or if there was a difference of ≥20% drop‐out rate between groups, and in these cases, it is possible that there is a plausible intervention effect among participants with missing data.

We endeavoured to identify unpublished studies by searching study registries and grey literature and contacting experts in the field, but we cannot rule out publication bias. We are not aware of unpublished studies with negative results, but given the nature of this research and the fact that many studies were funded or supported by industry, it is possible that smaller studies with nonsignificant results may be less likely to be published. Reporting of the content of the interventions was poor, and there were insufficient studies within each comparison to enable us to conduct a meta‐regression to identify the effective components of the interventions. There was no information reported on the cost‐effectiveness of these programmes or the costs to individuals compared with diets based on conventional food.

This review expands on the indicative findings of a previous review.^12^ By including more studies, and reducing the confidence interval around the point estimate, the present findings demonstrate that the weight loss at 1 year in interventions that include MR products is statistically significantly greater than in interventions that do not use these products. Other recent reviews have included weight loss interventions incorporating meal replacements.^49^, ^51^ but these reviews have considered the use of meal replacements alongside other weight loss interventions, and have therefore been unable to determine the specific effect of meal replacements per se. TDR have also been reviewed in various populations.^10^, ^11^, ^52^ Although TDR products are like MR in that they are designed to replace usual food‐based meals, TDR products are formulated to be used as the sole source of nutrition, that is, to replace all food in the diet. MR aims to replace one or more usual meals, but the instructions for use suggest eating a food‐based meal at least once a day to ensure adequate intake of nutrients and vitamins. MR can be purchased over the counter and used without guidance from a health professional, whereas weight loss programmes incorporating TDR are advised to be undertaken under supervision. Given these differences between MR and TDR programmes, it is important to consider their effectiveness independently.

The key finding of this review is that participants assigned to a MR diet compared with a diet only approach (akin to a self‐directed weight loss attempt) lose an additional 1.44 kg at 1 year, and this difference appears to be maintained up to 4 years. Likewise, many people join behavioural weight loss programmes to provide support to lose weight. The results of the present study suggest that all other things being equal, incorporating meal replacement products into such behavioural weight loss programmes enhances their effect (reflected in the MR diet + support versus diet + support comparison), with a 2.22 kg greater weight loss at 1 year in the MR group. Although these differences may appear to be modest, currently around 70% of the UK population with a BMI of 30 kg/m^2^ are trying to lose weight,^53^ and a sustained weight loss of even 1 kg will bring substantial benefits to public health.^54^, ^55^ Moreover, since the comparative interventions also lead to weight loss, by and large incorporating MR into weight loss programmes increases the proportion of participants who achieve ≥5% and ≥10% weight loss at 1 year.

Meal replacement products are consumer food products (as opposed to medical devices or drugs), widely available to be purchased over the counter without prescription, but at present are infrequently used by those attempting weight loss in their weight loss attempts.^56^ Advice and guidance provided by clinicians to encourage their use could deliver benefits at minimal cost to health care providers. Few studies included in the review reported adverse events, including the impact on the nutritional quality of the data. Two studies found no evidence of adverse events arising from the use of these products,^26^, ^41^ and five studies report that they may improve the overall nutritional quality of the diet.^5^, ^6^, ^18^, ^19^, ^35^

The greater weight loss observed in programmes incorporating MR suggests that this approach makes it easier to adhere to a reduced energy diet. However, there has been little detailed study of eating behaviours. It is possible that these fixed‐energy, portion‐controlled, or prepacked foods contain less energy than most self‐selected meals and snacks, or that the structure and external control associated with their use facilitate adherence. Further work could usefully examine the behavioural processes that facilitate weight loss which would be of use when designing effective weight loss programmes to enhance effectiveness and reach.

These findings provide important new evidence to inform clinical guidelines. At present in many countries, clinical recommendations for the treatment of obesity advise that individuals attempting weight loss should be advised to aim for an energy deficit of 2092 to 4184 kJ/day (500‐1000 kcal), but they do not suggest that MR could be used to help individuals achieve this deficit nor do they recommend MR as an effective dietary strategy for weight loss.^13^, ^15^ Guidelines in some countries do refer to meal replacement programmes. In the United States, the guidelines state that the strength of evidence on the longer term effect on weight of MR is low.^57^ Singaporean guidelines note that meal replacements may induce greater acute reductions in weight but are not advised for the long‐term management of overweight and obesity.^17^ Australian guidelines do not recommend meal replacements for weight loss beyond their use as part of a VLED.^16^

In conclusion, this review provides evidence that MR is an effective intervention for the treatment of overweight and obesity at a year, especially when used together with a support programme. MR could be recommended for inclusion in weight management programmes offered by professionals or as part of a self‐management strategy for people with overweight or obesity.

## CONFLICTS OF INTEREST

NMA, PA, and SAJ are investigators on a research grant from Cambridge Weight Plan UK Ltd. PA and SAJ are investigators on a research grant from Weight Watchers Inc.

## Supporting information


**Table S1**: Example of search strategy (MEDLINE)
**Table S2:** Descriptions of the intervention and control groups in each of the comparisons
**Table S3:** Justification of studies judged at high or unclear risk of bias in one or more domain
**Figure S1:** Forest plot of mean change in fasting blood glucose concentrations (mmol/L) from baseline to 1 year between interventions incorportaing meal replacments (MR) and control interventions
**Figure S2:** Forest plot of mean change in fasting serum insulin concentrations (pmol/L) from baseline to 1 year between interventions incorportaing meal replacments (MR) and control interventions
**Figure S3:** Forest plot of mean change in HbA1c (%) from baseline to 1 year between interventions incorportaing meal replacments (MR) and control interventions
**Figure S4:** Forest plot of mean change in total cholesterol concentrations (mmol/L) from baseline to 1 year between interventions incorportaing meal replacments (MR) and control interventions
**Figure S5:** Forest plot of mean change in LDL cholesterol concentrations (mmol/L) to 1 year between interventions incorportaing meal replacments (MR) and control interventions
**Figure S6:** Forest plot of mean change in HDL cholesterol concentrations (mmol/L) from baseline to 1 year between interventions incorportaing meal replacments (MR) and control interventions
**Figure S7:** Forest plot of mean change in triglyceride concentrations (mmol/L) from baseline to 1 year between interventions incorportaing meal replacments (MR) and control interventions
**Figure S8:** Forest plot of mean change in systolic blood pressure (mmHg) from baseline to 1 year between interventions incorportaing meal replacments (MR) and control interventions
**Figure S9:** Forest plot of mean change in diastolic blood pressure (mmHg) from baseline to 1 year between interventions incorportaing meal replacments (MR) and control interventions
