# Supplementary material for: A systematic review and meta‐analysis of the effectiveness of meal replacements for weight loss
Source: Obes Rev. 2019 Jan 24;20(4):569–87. doi: 10.1111/obr.12816 (PMC6849863; doi:10.1111/obr.12816)
Supplement: Supplementary file 1 — Table S1: Example of search strategy (MEDLINE) Table S2: Descriptions of the intervention and control groups in each of the comparisons Table S3: Justification of studies judged at high or unclear risk of bias in one or more domain Figure S1: Forest plot of mean change in fasting blood glucose concentrations (mmol/L) from baseline to 1 year between interventions incorportaing meal replacments (MR) and control interventions Figure S2: Forest plot of mean change in fasting serum insulin concentrations (pmol/L) from baseline to 1 year between interventions incorportaing meal replacments (MR) and control interventions Figure S3: Forest plot of mean change in HbA1c (%) from baseline to 1 year between interventions incorportaing meal replacments (MR) and control interventions Figure S4: Forest plot of mean change in total cholesterol concentrations (mmol/L) from baseline to 1 year between interventions incorportaing meal replacments (MR) and control interventions Figure S5: Forest plot of mean change in LDL cholesterol concentrations (mmol/L) to 1 year between interventions incorportaing meal replacments (MR) and control interventions Figure S6: Forest plot of mean change in HDL cholesterol concentrations (mmol/L) from baseline to 1 year between interventions incorportaing meal replacments (MR) and control interventions Figure S7: Forest plot of mean change in triglyceride concentrations (mmol/L) from baseline to 1 year between interventions incorportaing meal replacments (MR) and control interventions Figure S8: Forest plot of mean change in systolic blood pressure (mmHg) from baseline to 1 year between interventions incorportaing meal replacments (MR) and control interventions Figure S9: Forest plot of mean change in diastolic blood pressure (mmHg) from baseline to 1 year between interventions incorportaing meal replacments (MR) and control interventions [file OBR-20-569-s001.pdf]

Supplementary Table 1: Medline search strategy

Supplementary Table 1: Example of search strategy (MEDLINE) **2**

Supplementary Table 2: Descriptions of the intervention and control groups in each of the comparisons **3**

Supplementary Table 3: Justification of studies judged at high or unclear risk of bias in one or more domain **4**

Supplementary Figure 1: Forest plot of mean change in fasting blood glucose concentrations (mmol/L) from baseline to 1 year between interventions incorportaing meal replacments (MR) and control interventions **5**

Supplementary Figure 2: Forest plot of mean change in fasting serum insulin concentrations (pmol/L) from baseline to 1 year between interventions incorportaing meal replacments (MR) and control interventions **6**

Supplementary Figure 3: Forest plot of mean change in HbA1c (%) from baseline to 1 year between interventions incorportaing meal replacments (MR) and control interventions **7**

Supplementary Figure 4: Forest plot of mean change in total cholesterol concentrations (mmol/L) from baseline to 1 year between interventions incorportaing meal replacments (MR) and control interventions **8**

Supplementary Figure 5: Forest plot of mean change in LDL cholesterol concentrations (mmol/L) to 1 year between interventions incorportaing meal replacments (MR) and control interventions **9**

Supplementary Figure 6: Forest plot of mean change in HDL cholesterol concentrations (mmol/L) from baseline to 1 year between interventions incorportaing meal replacments (MR) and control interventions **10**

Supplementary Figure 7: Forest plot of mean change in triglyceride concentrations (mmol/L) from baseline to 1 year between interventions incorportaing meal replacments (MR) and control interventions **11**

Supplementary Table 1: Medline search strategy

Supplementary Figure 8: Forest plot of mean change in systolic blood pressure (mmHg) from baseline to 1 year between interventions incorportaing meal replacments (MR) and control interventions **12**

Supplementary Figure 9: Forest plot of mean change in diastolic blood pressure (mmHg) from baseline to 1 year between interventions incorportaing meal replacments (MR) and control interventions **13**

Supplementary Table 1: Medline search strategy

# ▲	Searches
1	overweight/ or exp obesity/
2	(obes* or overweight).ti,ab.
3	1 or 2
4	(meal? adj2 replace*).ti,ab.
5	((preprepared or pre-prepared or prepared) adj2 (meal? or food? or snack? or portion?)).ti,ab.
6	((prepack* or pre-pack*) adj2 (meal? or food? or snack? or portion?)).ti,ab.
7	(eating plan? or meal plan* or planned meal? or planned menu?).ti,ab.
8	(portion controlled adj2 (diet? or meal? or food? or weight loss)).ti,ab.
9	(liquid adj2 (diet? or meal? or food?)).ti,ab.
10	(replace* adj (snack? or drink? or food? or liquid?)).ti,ab.
11	shake?.ti,ab.
12	(commercial* adj2 (program* or intervention? or weight loss or diet?)).ti,ab.
13	(isoenergetic adj (diet? or food? or meal?)).ti,ab.
14	(jenny craig or diet chef or nutrisystem or slimfast or slim fast or medifast or optifast or hmr).ti,ab,in.
15	4 or 5 or 6 or 7 or 8 or 9 or 10 or 11 or 12 or 13 or 14
16	Weight Loss/
17	(weight adj3 (loss or lose or lost or losing or chang* or reduc* or maintain* or maintenance)).ti,ab.
18	body mass index/ or waist-hip ratio/ or Waist Circumference/
19	(bmi or body mass or waist-hip or waist circumference).ti,ab.
20	16 or 17 or 18 or 19
21	3 and 15 and 20
22	Weight Reduction Programs/
23	caloric restriction/ or diet, carbohydrate-restricted/ or diet, fat-restricted/ or diet, mediterranean/ or diet, reducing/
24	22 or 23
25	15 and 24
26	21 or 25
27	limit 26 to "reviews (maximizes specificity)"
28	randomized controlled trial.pt.
29	controlled clinical trial.pt.
30	randomized.ab.
31	placebo.ab.
32	drug therapy.fs.
33	randomly.ab.
34	trial.ab.
35	groups.ab.
36	28 or 29 or 30 or 31 or 32 or 33 or 34 or 35
37	exp animals/ not humans.sh.
38	36 not 37
39	26 and 38
40	27 or 39

Supplementary Table 2: Descriptions of the intervention and control groups in each of the comparisons

**Intervention arms**	Description	Example
MR diet	Guidance and/or provision of meal replacements to replace one or more daily meals or snacks. No additional provision of behavioural support programme.	Participants followed a self-selected diet, except that two of the three main meals (breakfast, lunch, dinner) were replaced with a liquid meal replacement [23].
MR diet + support	Programme of support provided (of similar intensity as the support provided in the control arm). This programme included guidance and/or provision of MR to replace one or more meals or snacks as per study specific protocol.	Participants were advised to follow a hypoenergetic diet consisting of 5 meal replacements and a self-select healthy meal.<br>They also met with a dietitian bi-weekly during for 16-weeks then twice during the 24 week weight maintenance phase for dietary and behavioural counselling [39].
MR diet + enhanced support	Guidance and/or provision of meal replacements alongside a programme of support based on behaviour change concepts which is of greater intensity to the support programme provided in the control arm.	Participants were advised to follow an individualised hypoenergetic diet that includes pre-packaged prepared food items that are accompanied by self-selected vegetables, fruit. Weekly interactions include strategies for making appropriate food choices and cognitive aspects of promoting weight loss and maintenance. In addition there was a goal of 30 minutes of physical activity on 5 or more days of the week [36].
**Control arms**		
Diet + support	Advice and guidance provided on weight loss	
Diet only	Intervention that specifies that only advice on dietary change, with no support to implement the dietary change was provided to participants.	Meal plan provided during initial contact with dietician, but no further contact
Minimal intervention	Minimal advice given	One-off contact, advice session written material

Supplementary Table 3: Justification of studies judged at high or unclear risk of bias in one or more domain

	Comment on judgement
**MR diet vs. diet-only**	
Ahrens 2003	Insufficient information on how randomisation sequence was generated.
	Insufficient information to determine if adequate allocation concealment was employed.
	Incomplete outcome data ; >50% missing
Cheskin 2008	Insufficient information to determine if adequate allocation concealment was employed.
	Unbalanced missing outcome data between groups
Ditschuneit 2001	Insufficient information to determine if adequate allocation concealment was employed.
Khoo 2011	Insufficient information on how randomisation sequence was generated.
	Insufficient information to determine if adequate allocation concealment was employed.
Rothacker 2001	Insufficient information on how randomisation sequence was generated.
	Insufficient information to determine if adequate allocation concealment was employed.
**MR diet + support vs. diet + support**	
Ashley 2001	Insufficient information on how randomisation sequence was generated.
	Insufficient information to determine if adequate allocation concealment was employed.
Ashley 2007	Insufficient information on how randomisation sequence was generated.
	Insufficient information to determine if adequate allocation concealment was employed.
Chee 2018	Insufficient information to determine if adequate allocation concealment was employed.
Davis 2010	Incomplete outcome data; >50% missing
Flechtner-Mors 2010	Unbalanced missing outcome data between groups
Li 2005	Unbalanced missing outcome data between groups
Lowe 2018	Insufficient information on how randomisation sequence was generated.
	Insufficient information to determine if adequate allocation concealment was employed.
Rolls 2017	Insufficient information to determine if adequate allocation concealment was employed.
**MR diet + support vs. diet only**	
Rock 2007	Insufficient information on how randomisation sequence was generated.
	Insufficient information to determine if adequate allocation concealment was employed.
**MR diet + enhanced support vs diet + support**	
Rock 2010	Insufficient information on how randomisation sequence was generated.
	Insufficient information to determine if adequate allocation concealment was employed.
Rock 2014	Insufficient information to determine if adequate allocation concealment was employed.
**MR diet + support vs. minimal control**	
Xu 2013	Insufficient information on how randomisation sequence was generated.
	Insufficient information to determine if adequate allocation concealment was employed.

Supplementary Figure 1: Forest plot of mean change in fasting blood glucose concentrations (mmol/L) from baseline to 1 year between interventions incorportaing meal replacments (MR) and control interventions

	Experimental			Control				Mean Difference
**Study or Subgroup**	**Mean [mmol/L]**	**SD [mmol/L]**	**Total**	**Mean [mmol/L]**	**SD [mmol/L]**	**Total**	**Weight**	**IV, Random, 95% CI [mmol/L]**
**2.1.1 MR diet vs diet only**								
Cheskin 2008	-1.14	1.44	54	-0.09	1.86	58	20.5%	-1.05 [-1.66, -0.44]
Ditschuneit 2001	-0.2	0.4	50	-0.23	0.44	50	33.9%	0.03 [-0.13, 0.19]
Khoo 2011	-0.11	0.78	19	-0.74	1.25	12	16.1%	0.63 [-0.16, 1.42]
Khoo 2013	-0.26	0.69	24	-0.14	0.43	24	29.5%	-0.12 [-0.45, 0.21]
**Subtotal (95% CI)**			**147**			**144**	**100.0%**	**-0.14 [-0.57, 0.29]**
Heterogeneity: Tau² = 0.13; Chi² = 14.09, df = 3 (P = 0.003); I² = 79%								
Test for overall effect: Z = 0.64 (P = 0.52)								
**2.1.2 MR diet + support vs diet + support**								
Flechtner-Mors 2010	-0.3	0.8	55	-0.5	1	55	27.4%	0.20 [-0.14, 0.54]
Li 2005	-0.6	3	52	-0.3	2.6	52	3.7%	-0.30 [-1.38, 0.78]
Rolls 2017	-0.2	0.4	60	-0.1	0.3	62	68.9%	-0.10 [-0.23, 0.03]
**Subtotal (95% CI)**			**167**			**169**	**100.0%**	**-0.03 [-0.24, 0.19]**
Heterogeneity: Tau² = 0.01; Chi² = 2.83, df = 2 (P = 0.24); I² = 29%								
Test for overall effect: Z = 0.23 (P = 0.82)								
**2.1.3 MR diet + support vs diet only**								
Shikany 2013	-0.12	0.34	60	-0.05	0.37	60	100.0%	-0.07 [-0.20, 0.06]
**Subtotal (95% CI)**			**60**			**60**	**100.0%**	**-0.07 [-0.20, 0.06]**
Heterogeneity: Not applicable								
Test for overall effect: Z = 1.08 (P = 0.28)								
**2.1.4 MR diet + enhanced support vs diet + support**								
Rock 2014 (1)	-0.7	1.5	77	0.7	1.7	38	50.6%	-1.40 [-2.04, -0.76]
Rock 2014 (2)	0.2	1.7	74	0.7	1.7	38	49.4%	-0.50 [-1.16, 0.16]
**Subtotal (95% CI)**			**151**			**76**	**100.0%**	**-0.96 [-1.84, -0.07]**
Heterogeneity: Tau² = 0.29; Chi² = 3.68, df = 1 (P = 0.06); I² = 73%								
Test for overall effect: Z = 2.12 (P = 0.03)								
**2.1.5 MR diet + support vs minimal control**								
Kempf 2018 (3)	-0.9	1.5	149	-0.2	1.5	50	15.2%	-0.70 [-1.18, -0.22]
Kempf 2018 (4)	-0.6	1.3	160	-0.2	1.5	50	15.9%	-0.40 [-0.86, 0.06]
LookAhead Research Group 2007	-1.16	0.2	2750	-0.38	0.083	2575	34.6%	-0.78 [-0.79, -0.77]
Xu 2013	-0.11	0.1	46	0.36	0.12	42	34.3%	-0.47 [-0.52, -0.42]
**Subtotal (95% CI)**			**3105**			**2717**	**100.0%**	**-0.60 [-0.85, -0.35]**
Heterogeneity: Tau² = 0.05; Chi² = 168.97, df = 3 (P < 0.00001); I² = 98%								
Test for overall effect: Z = 4.71 (P < 0.00001)								

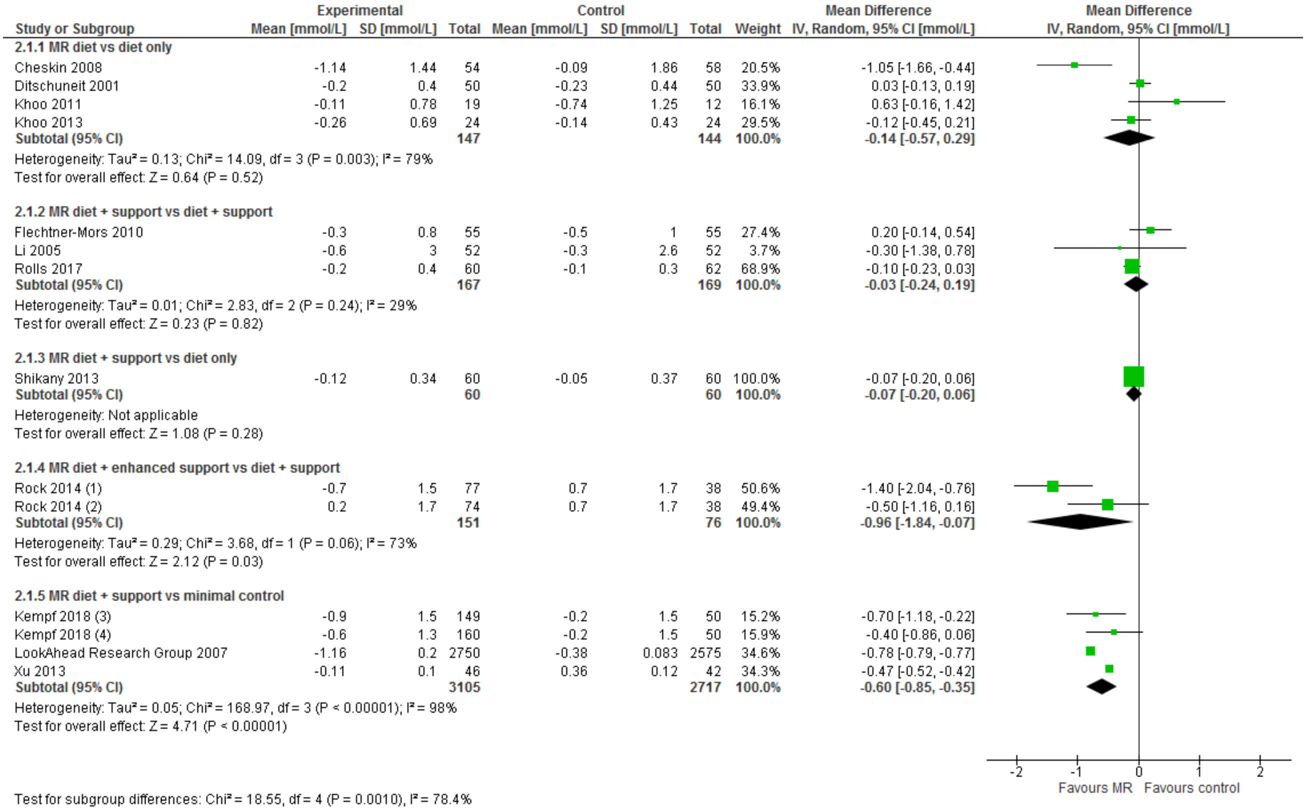

Test for subgroup differences: Chi² = 18.55, df = 4 (P = 0.0010), I² = 78.4%

Footnotes
(1) low carbohydrate MR
(2) low fat MR
(3) Stringent use of MR
(4) Moderate use of MR

Supplementary Figure 2: Forest plot of mean change in fasting serum insulin concentrations (pmol/L) from baseline to 1 year between interventions incorporating meal replacments (MR) and control interventions

	MR			Control				Mean Difference
Study or Subgroup	Mean [pmol/L]	SD [pmol/L]	Total	Mean [pmol/L]	SD [pmol/L]	Total	Weight	IV, Fixed, 95% CI [pmol/L]
**2.2.1 MR diet vs diet only**								
Cheskin 2008	-35.4	38.1	54	-0.9	31.9	58	18.1%	-34.50 [-47.56, -21.44]
Ditschuneit 2001	-33	26.9	50	-32.7	33.3	50	21.9%	-0.30 [-12.17, 11.57]
Khoo 2011	-13.6	35.4	19	48.34	19.6	12	8.2%	-61.94 [-81.34, -42.54]
Khoo 2013	-13.8	13.8	24	-11.9	13.5	24	51.8%	-1.90 [-9.62, 5.82]
**Subtotal (95% CI)**			**147**			**144**	**100.0%**	**-12.37 [-17.93, -6.82]**
Heterogeneity: Chi² = 47.14, df = 3 (P < 0.00001); I² = 94%								
Test for overall effect: Z = 4.36 (P < 0.0001)								
**2.2.2 MR diet + support vs diet + support**								
Flechtner-Mors 2010	-41.2	53.7	55	-96.6	97.6	55	0.1%	55.40 [25.96, 84.84]
Li 2005	-2.9	1.34	52	4.17	2.8	52	98.7%	-7.07 [-7.91, -6.23]
Rolls 2017	-4.167	22.2	62	-6.9	21.5	62	1.2%	2.73 [-4.96, 10.43]
**Subtotal (95% CI)**			**169**			**169**	**100.0%**	**-6.90 [-7.74, -6.06]**
Heterogeneity: Chi² = 23.38, df = 2 (P < 0.00001); I² = 91%								
Test for overall effect: Z = 16.14 (P < 0.00001)								
**2.2.3 MR diet + support vs diet only**								
Rock 2007	-25	38.9	35	11.8	29.9	35	100.0%	-36.80 [-53.05, -20.55]
**Subtotal (95% CI)**			**35**			**35**	**100.0%**	**-36.80 [-53.05, -20.55]**
Heterogeneity: Not applicable								
Test for overall effect: Z = 4.44 (P < 0.00001)								
**2.2.4 MR diet + enhanced support vs diet + support**								
Rock 2014 (1)	-62.5	65.5	77	-47.5	173.3	38	53.2%	-15.00 [-72.01, 42.01]
Rock 2014 (2)	-27.8	112.4	74	-47.5	173.3	38	46.8%	19.70 [-41.06, 80.46]
**Subtotal (95% CI)**			**151**			**76**	**100.0%**	**1.25 [-40.33, 42.82]**
Heterogeneity: Chi² = 0.67, df = 1 (P = 0.41); I² = 0%								
Test for overall effect: Z = 0.06 (P = 0.95)								
**2.2.5 MR diet + support vs minimal control**								
**Subtotal (95% CI)**			**0**			**0**		**Not estimable**
Heterogeneity: Not applicable								
Test for overall effect: Not applicable								

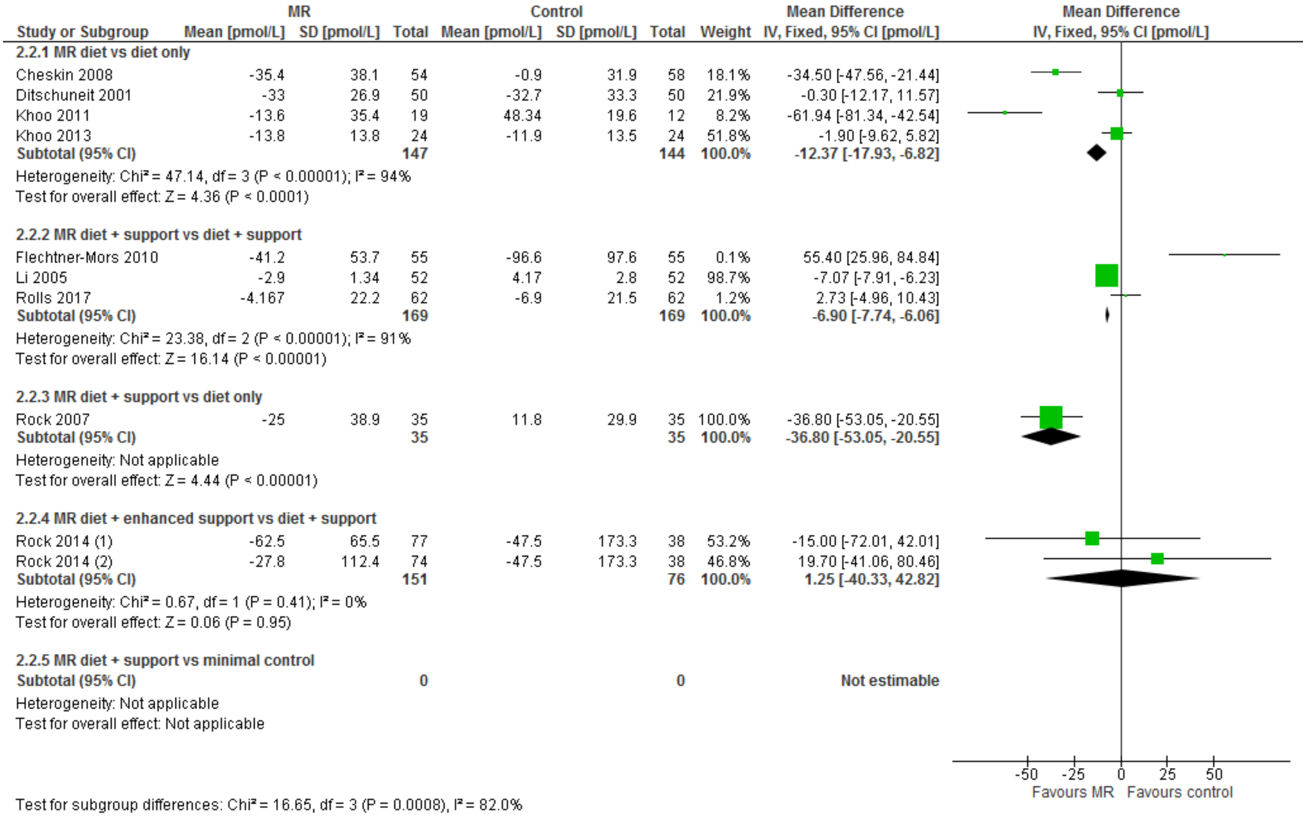

Test for subgroup differences: Chi² = 16.65, df = 3 (P = 0.0008), I² = 82.0%

Footnotes
(1) Low Fat MR
(2) Low CHO MR

Supplementary Figure 3: Forest plot of mean change in HbA1c (%) from baseline to 1 year between interventions incorporating meal replacments (MR) and control interventions

	MR			Control				Mean Difference
**Study or Subgroup**	**Mean [%]**	**SD [%]**	**Total**	**Mean [%]**	**SD [%]**	**Total**	**Weight**	**IV, Fixed, 95% CI [%]**
**2.3.1 MR diet vs diet only**								
Cheskin 2008	-0.6	0.9	54	0.2	1.2	58	100.0%	-0.80 [-1.19, -0.41]
**Subtotal (95% CI)**			**54**			**58**	**100.0%**	**-0.80 [-1.19, -0.41]**
Heterogeneity: Not applicable								
Test for overall effect: Z = 4.01 (P < 0.0001)								
**2.3.2 MR diet + support vs diet + support**								
Flechtner-Mors 2010	-0.1	0.2	55	-0.1	0.4	55	9.6%	0.00 [-0.12, 0.12]
Li 2005	-0.3	0.1	52	-0.1	0.1	52	90.4%	-0.20 [-0.24, -0.16]
**Subtotal (95% CI)**			**107**			**107**	**100.0%**	**-0.18 [-0.22, -0.14]**
Heterogeneity: Chi² = 9.95, df = 1 (P = 0.002); I² = 90%								
Test for overall effect: Z = 9.70 (P < 0.00001)								
**2.3.3 MR diet + support vs diet only**								
**Subtotal (95% CI)**			**0**			**0**		**Not estimable**
Heterogeneity: Not applicable								
Test for overall effect: Not applicable								
**2.3.4 MR diet + enhanced support vs diet + support**								
Rock 2014 (1)	-0.3	0.7	74	0.1	0.7	38	49.7%	-0.40 [-0.67, -0.13]
Rock 2014 (2)	-0.6	0.7	77	0.1	0.7	38	50.3%	-0.70 [-0.97, -0.43]
**Subtotal (95% CI)**			**151**			**76**	**100.0%**	**-0.55 [-0.74, -0.36]**
Heterogeneity: Chi² = 2.32, df = 1 (P = 0.13); I² = 57%								
Test for overall effect: Z = 5.60 (P < 0.00001)								
**2.3.5 MR diet + support vs minimal control**								
Kempf 2018 (3)	-0.4	0.6	160	0	0.5	50	2.7%	-0.40 [-0.57, -0.23]
Kempf 2018 (4)	-0.6	0.6	149	0	0.5	50	2.6%	-0.60 [-0.77, -0.43]
LookAhead Research Group 2007	-0.6	0.5	2750	-0.1	0.56	2575	91.8%	-0.50 [-0.53, -0.47]
Xu 2013	-0.1	0.2	46	0.1	0.5	42	2.9%	-0.20 [-0.36, -0.04]
**Subtotal (95% CI)**			**3105**			**2717**	**100.0%**	**-0.49 [-0.52, -0.46]**
Heterogeneity: Chi² = 15.54, df = 3 (P = 0.001); I² = 81%								
Test for overall effect: Z = 35.16 (P < 0.00001)								

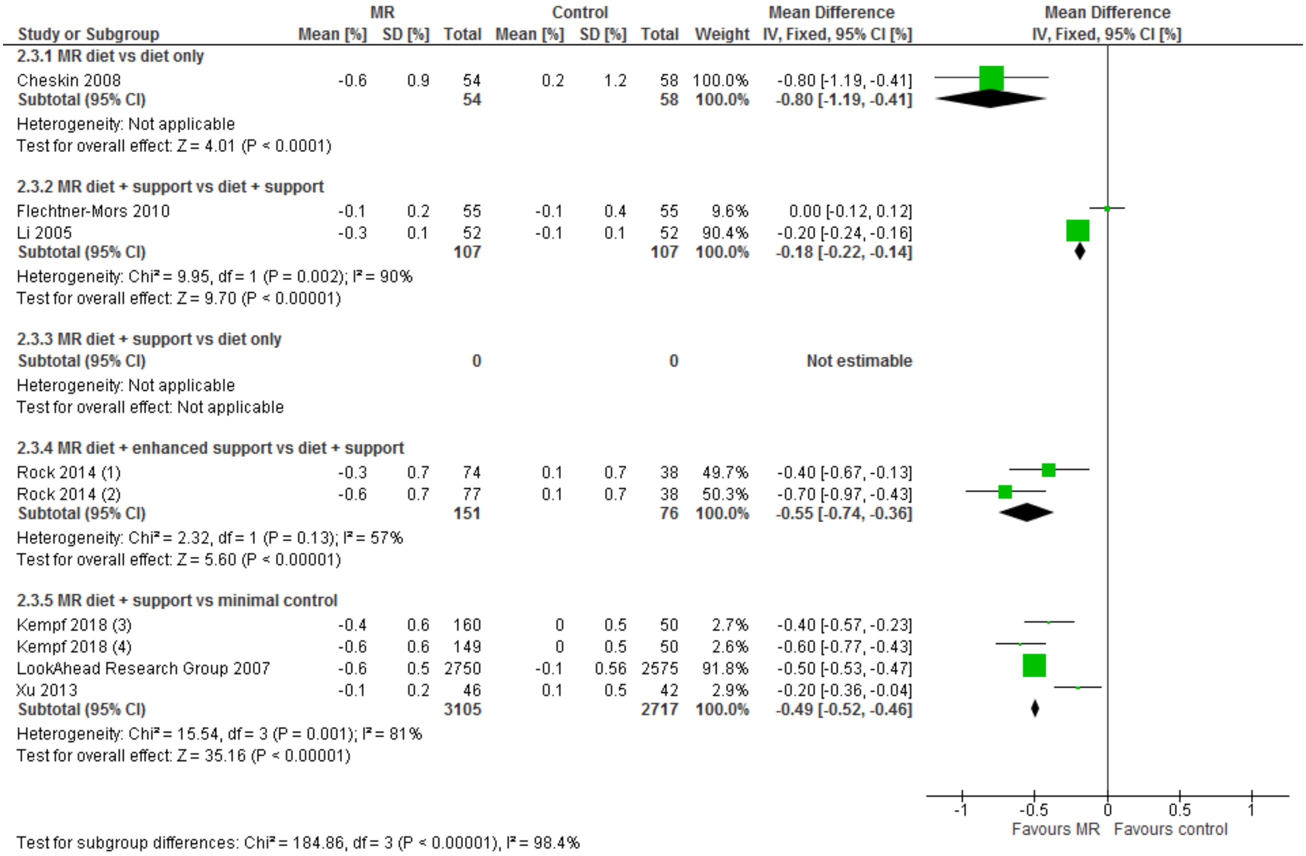

Test for subgroup differences: Chi² = 184.86, df = 3 (P < 0.00001), I² = 98.4%

Footnotes
(1) low fat MR
(2) low cho MR
(3) Moderate use of MR
(4) Stringent use of MR

Supplementary Figure 4: Forest plot of mean change in total cholesterol concentrations (mmol/L) from baseline to 1 year between interventions incorporating meal replacments (MR) and control interventions

	Experimental			Control				Mean Difference
**Study or Subgroup**	**Mean [mmol/L]**	**SD [mmol/L]**	**Total**	**Mean [mmol/L]**	**SD [mmol/L]**	**Total**	**Weight**	**IV, Random, 95% CI [mmol/L]**
**2.4.1 MR diet vs diet only**								
Cheskin 2008	-0.19	0.5	54	-0.05	0.48	58	15.7%	-0.14 [-0.32, 0.04]
Ditschuneit 2001	-0.1	0.2	50	0	0.2	50	84.3%	-0.10 [-0.18, -0.02]
**Subtotal (95% CI)**			**104**			**108**	**100.0%**	**-0.11 [-0.18, -0.03]**
Heterogeneity: Tau² = 0.00; Chi² = 0.16, df = 1 (P = 0.69); I² = 0%								
Test for overall effect: Z = 2.89 (P = 0.004)								
**2.4.2 MR diet + support vs diet + support**								
Ashley 2001 (1)	-0.3	0.3	38	-0.1	0.4	18	6.5%	-0.20 [-0.41, 0.01]
Ashley 2001 (2)	-0.2	0.8	38	-0.1	0.4	19	3.1%	-0.10 [-0.41, 0.21]
Davis 2010	-0.12	0.37	45	-0.05	0.37	45	11.1%	-0.07 [-0.22, 0.08]
Flechtner-Mors 2010	-0.07	0.35	55	-0.09	0.43	55	11.9%	0.02 [-0.13, 0.17]
Li 2005	0.003	0.08	52	0.1	0.06	52	55.8%	-0.10 [-0.12, -0.07]
Rolls 2017	0.05	0.44	62	0.01	0.4	62	11.7%	0.04 [-0.11, 0.19]
**Subtotal (95% CI)**			**290**			**251**	**100.0%**	**-0.07 [-0.13, -0.01]**
Heterogeneity: Tau² = 0.00; Chi² = 6.53, df = 5 (P = 0.26); I² = 23%								
Test for overall effect: Z = 2.48 (P = 0.01)								
**2.4.3 MR diet + support vs diet only**								
Rock 2007	-0.02	0.44	35	0.06	0.47	35	36.1%	-0.08 [-0.29, 0.13]
Shikany 2013	0.01	0.39	60	0.04	0.5	60	63.9%	-0.03 [-0.19, 0.13]
**Subtotal (95% CI)**			**95**			**95**	**100.0%**	**-0.05 [-0.18, 0.08]**
Heterogeneity: Tau² = 0.00; Chi² = 0.13, df = 1 (P = 0.71); I² = 0%								
Test for overall effect: Z = 0.73 (P = 0.46)								
**2.4.4 MR diet + enhanced support vs diet + support**								
Rock 2010 (3)	-0.14	0.46	167	-0.17	0.39	56	33.8%	0.03 [-0.09, 0.15]
Rock 2010 (4)	-0.14	0.44	164	-0.17	0.39	55	34.1%	0.03 [-0.09, 0.15]
Rock 2014 (5)	0.25	0.44	74	0.2	0.46	38	16.5%	0.05 [-0.13, 0.23]
Rock 2014 (6)	0.3	0.4	77	0.2	0.5	38	15.6%	0.10 [-0.08, 0.28]
**Subtotal (95% CI)**			**482**			**187**	**100.0%**	**0.04 [-0.03, 0.12]**
Heterogeneity: Tau² = 0.00; Chi² = 0.47, df = 3 (P = 0.93); I² = 0%								
Test for overall effect: Z = 1.20 (P = 0.23)								
**2.4.5 MR diet + support vs minimal control**								
Kempf 2018 (7)	0	0.53	149	0.34	0.5	50	31.6%	-0.34 [-0.50, -0.18]
Kempf 2018 (8)	-0.1	0.53	162	0.34	0.5	50	31.7%	-0.44 [-0.60, -0.28]
Xu 2013	-0.1	0.1	46	0	0.1	42	36.8%	-0.10 [-0.14, -0.06]
**Subtotal (95% CI)**			**357**			**142**	**100.0%**	**-0.28 [-0.52, -0.05]**
Heterogeneity: Tau² = 0.04; Chi² = 22.61, df = 2 (P < 0.0001); I² = 91%								
Test for overall effect: Z = 2.37 (P = 0.02)								

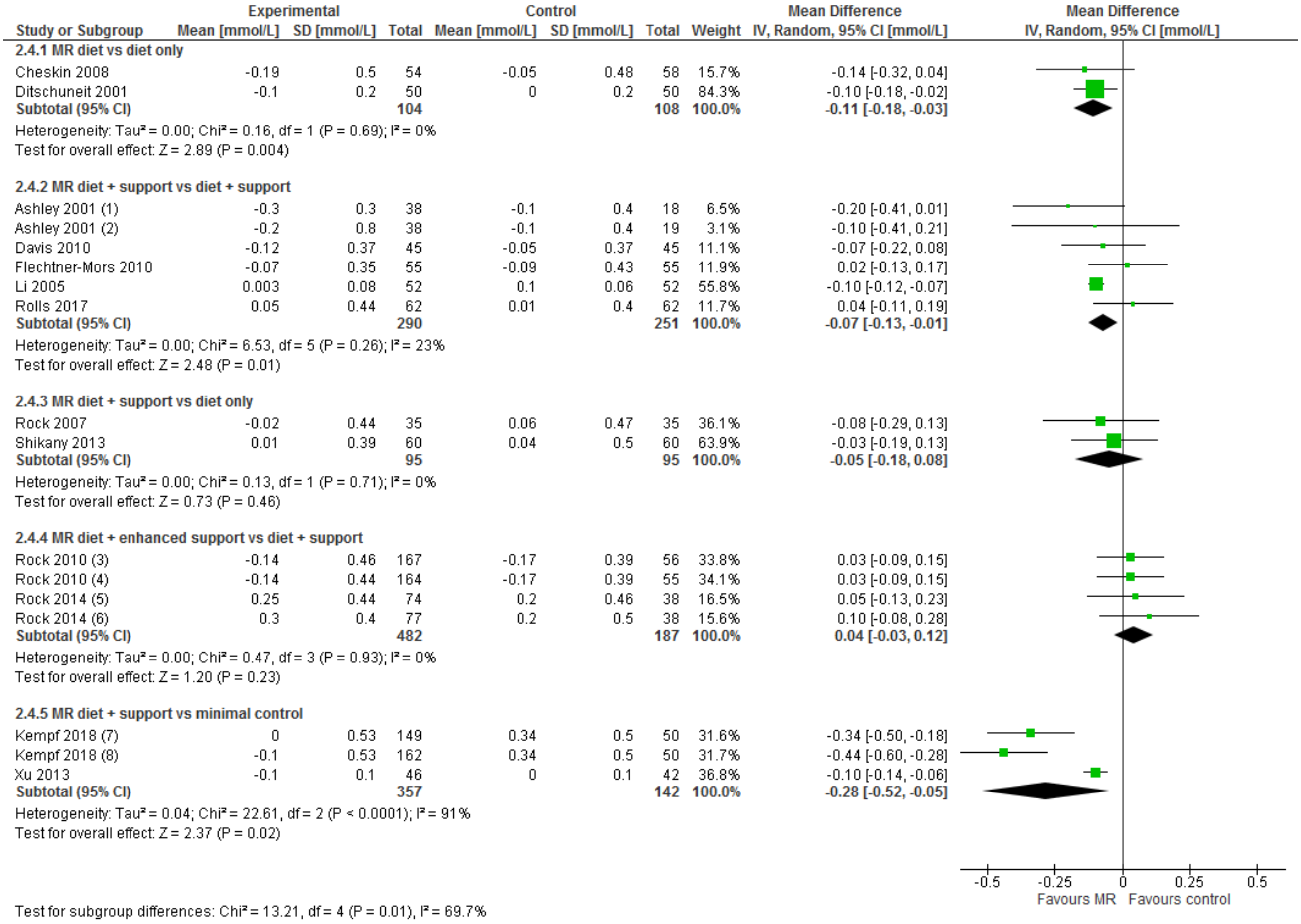

Test for subgroup differences: Chi² = 13.21, df = 4 (P = 0.01), I² = 69.7%

Footnotes
(1) physician/nurse led intervention
(2) dietician led intervention
(3) in person support
(4) telephone support
(5) low fat MR
(6) low carbohydrate MR
(7) Stringent use MR
(8) Moderate use MR

Supplementary Figure 5: Forest plot of mean change in LDL cholesterol concentrations (mmol/L) to 1 year between interventions incorportaing meal replacments (MR) and control interventions

	MR			Control				Mean Difference
Study or Subgroup	Mean [mmol/L]	SD [mmol/L]	Total	Mean [mmol/L]	SD [mmol/L]	Total	Weight	IV, Random, 95% CI [mmol/L]
**2.5.1 MR diet + diet only**								
Cheskin 2008	0	0.5	54	0.1	0.4	58	58.3%	-0.10 [-0.27, 0.07]
Khoo 2011	0.1	0.6	19	-0.3	0.6	12	41.7%	0.40 [-0.03, 0.83]
**Subtotal (95% CI)**			**73**			**70**	**100.0%**	**0.11 [-0.37, 0.59]**
Heterogeneity: Tau² = 0.10; Chi² = 4.44, df = 1 (P = 0.04); I² = 77%								
Test for overall effect: Z = 0.44 (P = 0.66)								
**2.5.2 MR diet + support vs diet + support**								
Ashley 2001 (1)	-0.13	0.47	38	-0.03	0.26	18	17.5%	-0.10 [-0.29, 0.09]
Ashley 2001 (2)	-0.21	0.57	38	-0.03	0.26	19	16.3%	-0.18 [-0.40, 0.04]
Davis 2010	-0.13	0.31	45	-0.05	0.28	45	21.1%	-0.08 [-0.20, 0.04]
Li 2005	-0.14	0.05	52	0.16	0.1	52	24.3%	-0.30 [-0.33, -0.27]
Rolls 2017	-0.05	0.39	60	-0.05	0.35	62	20.7%	0.00 [-0.13, 0.13]
**Subtotal (95% CI)**			**233**			**196**	**100.0%**	**-0.14 [-0.29, 0.01]**
Heterogeneity: Tau² = 0.02; Chi² = 32.89, df = 4 (P < 0.00001); I² = 88%								
Test for overall effect: Z = 1.79 (P = 0.07)								
**2.5.3 MR diet + support vs diet only**								
Rock 2007	-0.2	0.5	35	0	0.4	35	42.7%	-0.20 [-0.41, 0.01]
Shikany 2013	-0.03	0.36	60	-0.04	0.43	60	57.3%	0.01 [-0.13, 0.15]
**Subtotal (95% CI)**			**95**			**95**	**100.0%**	**-0.08 [-0.28, 0.12]**
Heterogeneity: Tau² = 0.01; Chi² = 2.60, df = 1 (P = 0.11); I² = 62%								
Test for overall effect: Z = 0.77 (P = 0.44)								
**2.5.4 MR diet + enhanced support vs diet + support**								
Rock 2010 (3)	-0.26	0.4	167	-0.17	0.37	56	31.6%	-0.09 [-0.20, 0.02]
Rock 2010 (4)	-0.07	1.89	164	-0.17	0.37	55	14.3%	0.10 [-0.21, 0.41]
Rock 2014 (5)	0.09	0.4	74	-0.04	0.4	38	27.0%	0.13 [-0.03, 0.29]
Rock 2014 (6)	0.13	0.4	77	-0.04	0.4	38	27.1%	0.17 [0.01, 0.33]
**Subtotal (95% CI)**			**482**			**187**	**100.0%**	**0.07 [-0.08, 0.21]**
Heterogeneity: Tau² = 0.01; Chi² = 9.07, df = 3 (P = 0.03); I² = 67%								
Test for overall effect: Z = 0.91 (P = 0.36)								
**2.5.5 MR diet + support vs minimal control**								
Kempf 2018 (7)	0.04	0.39	149	-0.04	0.39	50	24.6%	0.08 [-0.04, 0.20]
Kempf 2018 (8)	-0.13	0.35	160	-0.04	0.39	50	24.6%	-0.09 [-0.21, 0.03]
LookAhead Research Group 2007	-0.13	0.39	2570	-0.15	0.42	2575	25.4%	0.02 [-0.00, 0.04]
Xu 2013	-0.2	0.1	46	0.3	0.1	42	25.4%	-0.50 [-0.54, -0.46]
**Subtotal (95% CI)**			**2925**			**2717**	**100.0%**	**-0.12 [-0.45, 0.20]**
Heterogeneity: Tau² = 0.11; Chi² = 470.93, df = 3 (P < 0.00001); I² = 99%								
Test for overall effect: Z = 0.74 (P = 0.46)								

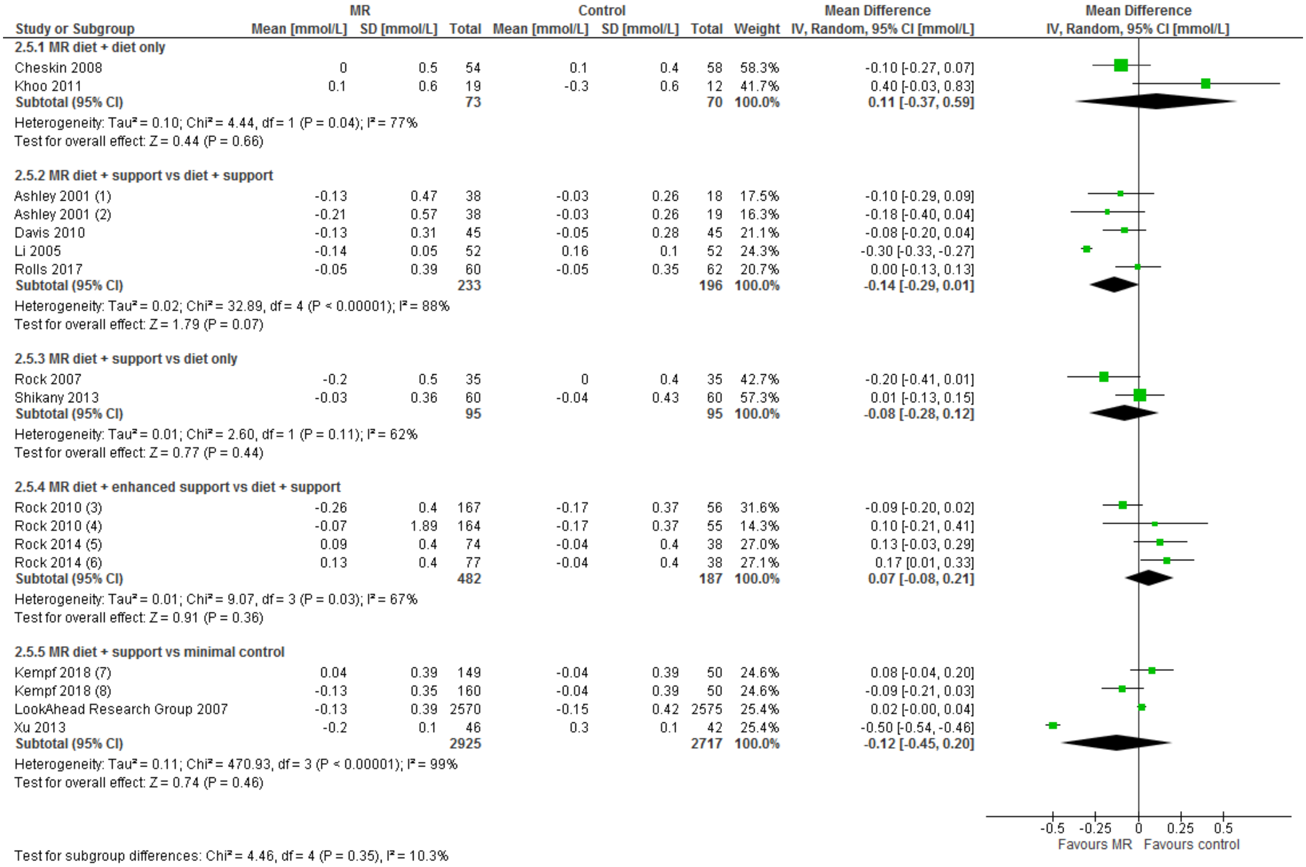

Test for subgroup differences: Chi² = 4.46, df = 4 (P = 0.35), I² = 10.3%

Footnotes
(1) physician/nurse led intervention
(2) dietician led intervention
(3) in person support
(4) telephone support
(5) low fat MR
(6) low carbohydrate MR
(7) Stringent MR use
(8) Moderate MR use

Supplementary Figure 6: Forest plot of mean change in HDL cholesterol concentrations (mmol/L) from baseline to 1 year between interventions incorporating meal replacments (MR) and control interventions

Study or Subgroup	Experimental Mean [mmol/L]	Experimental SD [mmol/L]	Experimental Total	Control Mean [mmol/L]	Control SD [mmol/L]	Control Total	Weight	Mean Difference IV, Random, 95% CI [mmol/L]
**2.6.1 MR diet vs diet only**								
Cheskin 2008	0	0.2	54	0	0.2	58	17.8%	0.00 [-0.07, 0.07]
Ditschuneit 2001	0	0.1	50	0	0.1	50	63.5%	0.00 [-0.04, 0.04]
Khoo 2011	0	0.1	19	0	0.1	12	18.7%	0.00 [-0.07, 0.07]
**Subtotal (95% CI)**			**123**			**120**	**100.0%**	**0.00 [-0.03, 0.03]**
Heterogeneity: Tau² = 0.00; Chi² = 0.00, df = 2 (P = 1.00); I² = 0%								
Test for overall effect: Z = 0.00 (P = 1.00)								
**2.6.2 MR diet + support vs support only**								
Davis 2010	0.02	0.14	45	-0.03	0.16	45	22.6%	0.05 [-0.01, 0.11]
Flechtner-Mors 2010	0.03	0.13	55	0	0.16	55	24.0%	0.03 [-0.02, 0.08]
Li 2005	-0.02	0.01	52	0.04	0.03	52	30.1%	-0.06 [-0.07, -0.05]
Rolls 2017	0.08	0.17	60	0.11	0.16	62	23.3%	-0.03 [-0.09, 0.03]
**Subtotal (95% CI)**			**212**			**214**	**100.0%**	**-0.01 [-0.07, 0.05]**
Heterogeneity: Tau² = 0.00; Chi² = 22.32, df = 3 (P < 0.0001); I² = 87%								
Test for overall effect: Z = 0.22 (P = 0.83)								
**2.6.3 MR diet + support vs diet only**								
Rock 2007	0.3	0.2	35	0.1	0.2	35	47.8%	0.20 [0.11, 0.29]
Shikany 2013	0.06	0.17	60	0.03	0.18	60	52.2%	0.03 [-0.03, 0.09]
**Subtotal (95% CI)**			**95**			**95**	**100.0%**	**0.11 [-0.06, 0.28]**
Heterogeneity: Tau² = 0.01; Chi² = 8.74, df = 1 (P = 0.003); I² = 89%								
Test for overall effect: Z = 1.31 (P = 0.19)								
**2.6.4 MR diet + enhanced support vs diet + support**								
Rock 2010 (1)	0.02	0.25	167	0.02	0.18	55	26.9%	0.00 [-0.06, 0.06]
Rock 2010 (2)	0.05	0.26	164	0.02	0.18	56	26.7%	0.03 [-0.03, 0.09]
Rock 2014 (3)	0.33	0.19	74	0.22	0.19	38	23.1%	0.11 [0.04, 0.18]
Rock 2014 (4)	0.33	0.19	77	0.22	0.19	38	23.3%	0.11 [0.04, 0.18]
**Subtotal (95% CI)**			**482**			**187**	**100.0%**	**0.06 [0.00, 0.11]**
Heterogeneity: Tau² = 0.00; Chi² = 8.00, df = 3 (P = 0.05); I² = 62%								
Test for overall effect: Z = 2.11 (P = 0.04)								
**2.6.5 MR diet + support vs. minimal control**								
Kempf 2018 (5)	0.08	0.6	149	0	0.14	50	0.8%	0.08 [-0.02, 0.18]
Kempf 2018 (6)	0.02	0.12	160	0	0.14	50	4.3%	0.02 [-0.02, 0.06]
LookAhead Research Group 2007	0.09	0.18	2570	0.03	0.14	2575	64.6%	0.06 [0.05, 0.07]
Xu 2013	0.04	0.03	46	-0.02	0.04	42	30.3%	0.06 [0.05, 0.07]
**Subtotal (95% CI)**			**2925**			**2717**	**100.0%**	**0.06 [0.05, 0.07]**
Heterogeneity: Tau² = 0.00; Chi² = 3.38, df = 3 (P = 0.34); I² = 11%								
Test for overall effect: Z = 12.64 (P < 0.00001)								

Mean Difference IV, Random, 95% CI [mmol/L]

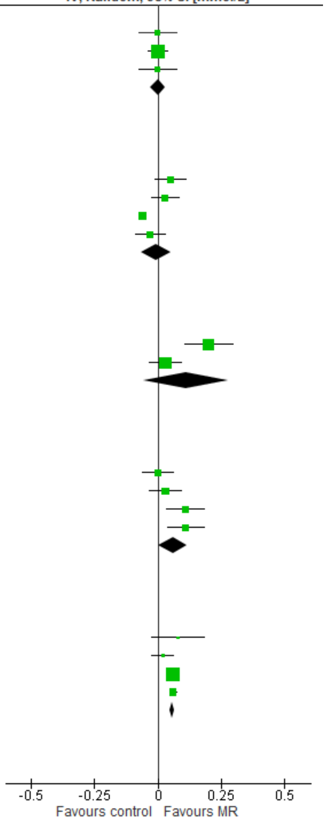

Test for subgroup differences: Chi² = 16.90, df = 4 (P = 0.002), I² = 76.3%

Footnotes
(1) in person support
(2) telephone support
(3) low fat MR
(4) low carbohydrate MR
(5) Stringent MR use
(6) Moderate MR use

Supplementary Figure 7: Forest plot of mean change in triglyceride concentrations (mmol/L) from baseline to 1 year between interventions incorportaing meal replacments (MR) and control interventions

Study or Subgroup	MR Mean [mmol/L]	MR SD [mmol/L]	MR Total	Control Mean [mmol/L]	Control SD [mmol/L]	Control Total	Weight	Mean Difference IV, Random, 95% CI [mmol/L]
**2.7.1 MR diet vs. diet only**								
Cheskin 2008	-0.4	0.6	54	0.1	1.1	58	33.0%	-0.50 [-0.83, -0.17]
Ditschuneit 2001	-0.1	0.4	50	-0.1	0.4	50	42.2%	0.00 [-0.16, 0.16]
Khoo 2011	-0.3	0.6	19	-0.4	0.7	12	24.8%	0.10 [-0.38, 0.58]
**Subtotal (95% CI)**			**123**			**120**	**100.0%**	**-0.14 [-0.49, 0.21]**
Heterogeneity: Tau² = 0.07; Chi² = 7.95, df = 2 (P = 0.02); I² = 75%								
Test for overall effect: Z = 0.78 (P = 0.43)								
**2.7.2 MR diet + support vs diet + support**								
Davis 2010	-0.01	0.27	45	0.01	0.37	45	29.6%	-0.02 [-0.15, 0.11]
Li 2005	-0.28	0.1	52	-0.23	0.15	52	41.5%	-0.05 [-0.10, -0.00]
Rolls 2017	0.04	0.28	60	-0.13	0.48	62	28.9%	0.17 [0.03, 0.31]
**Subtotal (95% CI)**			**157**			**159**	**100.0%**	**0.02 [-0.10, 0.15]**
Heterogeneity: Tau² = 0.01; Chi² = 8.58, df = 2 (P = 0.01); I² = 77%								
Test for overall effect: Z = 0.35 (P = 0.73)								
**2.7.3 MR diet + support vs diet only**								
Rock 2007	-0.1	0.3	35	0	0.3	35	38.4%	-0.10 [-0.24, 0.04]
Shikany 2013	-0.1	0.32	60	-0.02	0.3	60	61.6%	-0.08 [-0.19, 0.03]
**Subtotal (95% CI)**			**95**			**95**	**100.0%**	**-0.09 [-0.17, -0.00]**
Heterogeneity: Tau² = 0.00; Chi² = 0.05, df = 1 (P = 0.83); I² = 0%								
Test for overall effect: Z = 1.97 (P = 0.05)								
**2.7.4 MR diet + enhanced support vs diet + support**								
Rock 2010 (1)	-0.03	0.23	167	-0.04	0.26	56	28.7%	0.01 [-0.07, 0.09]
Rock 2010 (2)	-0.1	0.44	164	-0.04	0.26	55	28.1%	-0.06 [-0.16, 0.04]
Rock 2014 (3)	-0.16	0.5	74	0.22	0.64	38	21.7%	-0.38 [-0.61, -0.15]
Rock 2014 (4)	-0.37	0.54	77	0.22	0.64	38	21.5%	-0.59 [-0.83, -0.35]
**Subtotal (95% CI)**			**482**			**187**	**100.0%**	**-0.22 [-0.43, -0.02]**
Heterogeneity: Tau² = 0.04; Chi² = 29.59, df = 3 (P < 0.00001); I² = 90%								
Test for overall effect: Z = 2.11 (P = 0.04)								
**2.7.5 MR diet + support vs minimal control**								
LookAhead Research Group 2007	0.006	0.48	2570	0.145	0.496	2575	50.3%	-0.14 [-0.17, -0.11]
Xu 2013	-0.29	0.15	46	-0.44	0.07	42	49.7%	0.15 [0.10, 0.20]
**Subtotal (95% CI)**			**2616**			**2617**	**100.0%**	**0.00 [-0.28, 0.29]**
Heterogeneity: Tau² = 0.04; Chi² = 105.59, df = 1 (P < 0.00001); I² = 99%								
Test for overall effect: Z = 0.03 (P = 0.97)								

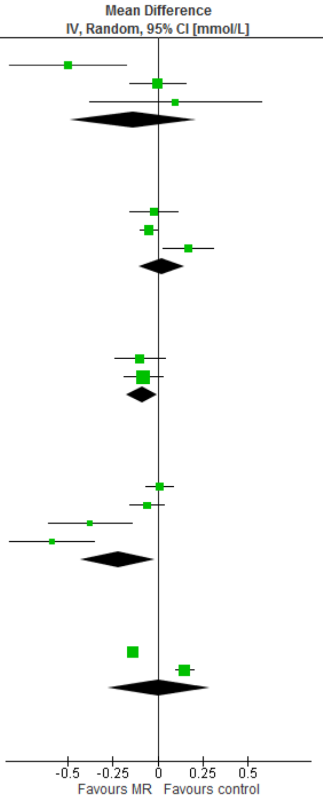

Test for subgroup differences: Chi² = 4.72, df = 4 (P = 0.32), I² = 15.3%

Footnotes
(1) in person support
(2) telephone support
(3) low fat MR
(4) low carbohydrate MR

Supplementary Figure 8: Forest plot of mean change in systolic blood pressure (mmHg) from baseline to 1 year between interventions incorportaing meal replacments (MR) and control interventions

	MR			Control				Mean Difference
Study or Subgroup	Mean [mmHg]	SD [mmHg]	Total	Mean [mmHg]	SD [mmHg]	Total	Weight	IV, Random, 95% CI [mmHg]
**2.8.1 MR diet vs. diet only**								
Cheskin 2008	-9.5	12	54	0.1	5.9	58	65.7%	-9.60 [-13.14, -6.06]
Ditschuneit 2001	-9.6	16.9	50	-3.8	10.2	50	34.3%	-5.80 [-11.27, -0.33]
**Subtotal (95% CI)**			**104**			**108**	**100.0%**	**-8.30 [-11.83, -4.76]**
Heterogeneity: Tau² = 1.69; Chi² = 1.31, df = 1 (P = 0.25); I² = 23%								
Test for overall effect: Z = 4.60 (P < 0.00001)								
**2.8.2 MR diet + support vs diet + support**								
Ashley 2001 (1)	-2.11	2.49	38	0.73	6.85	19	0.7%	-2.84 [-6.02, 0.34]
Ashley 2001 (2)	-1.45	6.16	38	0.73	6.85	18	0.5%	-2.18 [-5.90, 1.54]
Davis 2010	-4.74	6.54	45	-4.04	6.51	45	1.0%	-0.70 [-3.40, 2.00]
Flechtner-Mors 2010	-0.39	0.66	55	-0.09	0.81	55	96.4%	-0.30 [-0.58, -0.02]
Rolls 2017	-0.7	6.4	60	-0.6	7	62	1.3%	-0.10 [-2.48, 2.28]
**Subtotal (95% CI)**			**236**			**199**	**100.0%**	**-0.33 [-0.60, -0.06]**
Heterogeneity: Tau² = 0.00; Chi² = 3.50, df = 4 (P = 0.48); I² = 0%								
Test for overall effect: Z = 2.38 (P = 0.02)								
**2.8.3 MR diet + support vs. diet only**								
Shikany 2013	-0.67	6.79	60	0.93	5.78	60	100.0%	-1.60 [-3.86, 0.66]
**Subtotal (95% CI)**			**60**			**60**	**100.0%**	**-1.60 [-3.86, 0.66]**
Heterogeneity: Not applicable								
Test for overall effect: Z = 1.39 (P = 0.16)								
**2.8.4 MR diet + enhanced support vs diet + support**								
Rock 2014 (3)	-0.09	0.47	74	-0.43	0.76	38	49.7%	0.34 [0.08, 0.60]
Rock 2014 (4)	-0.09	0.46	77	-0.43	0.76	38	50.3%	0.34 [0.08, 0.60]
**Subtotal (95% CI)**			**151**			**76**	**100.0%**	**0.34 [0.15, 0.53]**
Heterogeneity: Tau² = 0.00; Chi² = 0.00, df = 1 (P = 1.00); I² = 0%								
Test for overall effect: Z = 3.58 (P = 0.0003)								
**2.8.5 MR diet + support vs minimal control**								
Kempf 2018 (5)	-4.2	7.5	160	-0.7	5.3	50	5.0%	-3.50 [-5.37, -1.63]
Kempf 2018 (6)	-4.5	6.3	149	-0.7	5.3	50	5.5%	-3.80 [-5.58, -2.02]
LookAhead Research Group 2007	-6.59	9.43	2570	-2.73	9.25	2575	67.1%	-3.86 [-4.37, -3.35]
Xu 2013	-2.3	2.4	46	1.2	1.8	42	22.5%	-3.50 [-4.38, -2.62]
**Subtotal (95% CI)**			**2925**			**2717**	**100.0%**	**-3.76 [-4.18, -3.34]**
Heterogeneity: Tau² = 0.00; Chi² = 0.56, df = 3 (P = 0.91); I² = 0%								
Test for overall effect: Z = 17.62 (P < 0.00001)								

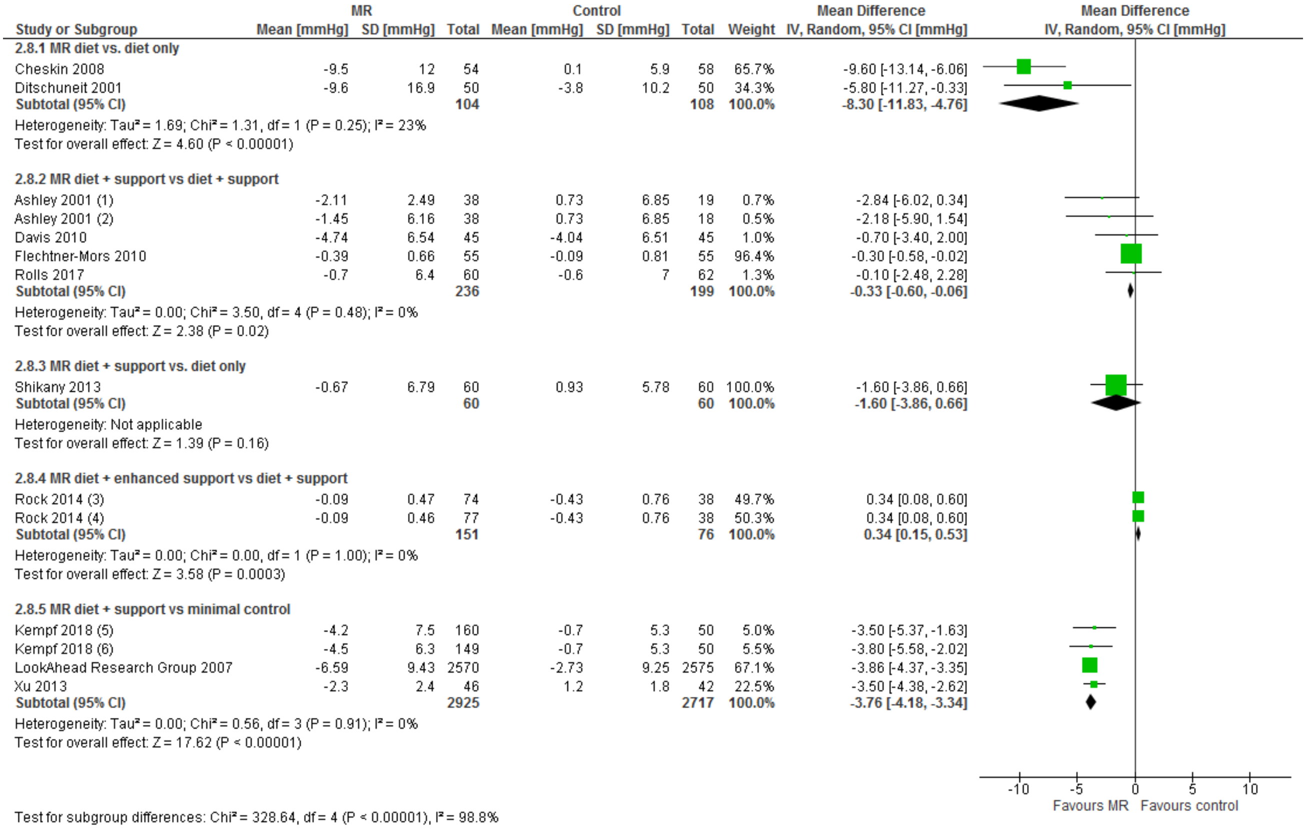

Test for subgroup differences: Chi² = 328.64, df = 4 (P < 0.00001), I² = 98.8%

Footnotes
(1) dietician led intervention
(2) physician/nurse led intervention
(3) low fat MR
(4) low carbohydrate MR
(5) Moderate MR use
(6) Stringent MR use

Supplementary Figure 9: Forest plot of mean change in diastolic blood pressure (mmHg) from baseline to 1 year between interventions incorporating meal replacements (MR) and control interventions

	MR			Control				Mean Difference
**Study or Subgroup**	**Mean [mmHg]**	**SD [mmHg]**	**Total**	**Mean [mmHg]**	**SD [mmHg]**	**Total**	**Weight**	**IV, Random, 95% CI [mmHg]**
**2.9.1 MR diet vs. diet only**								
Cheskin 2008	-5.2	7.2	54	-0.8	3.7	58	38.6%	-4.40 [-6.54, -2.26]
Ditschuneit 2001	-3.4	10.8	50	-3.1	3.5	50	27.2%	-0.30 [-3.45, 2.85]
Khoo 2013	-3.5	5	24	-0.5	3.7	24	34.2%	-3.00 [-5.49, -0.51]
**Subtotal (95% CI)**			**128**			**132**	**100.0%**	**-2.81 [-5.01, -0.60]**
Heterogeneity: Tau² = 2.09; Chi² = 4.46, df = 2 (P = 0.11); I² = 55%								
Test for overall effect: Z = 2.49 (P = 0.01)								
**2.9.2 MR diet + support vs. diet + support**								
Ashley 2001 (1)	-0.84	5.19	38	-0.32	4.39	19	18.2%	-0.52 [-3.09, 2.05]
Ashley 2001 (2)	-1.16	5.03	38	-0.32	4.39	18	18.1%	-0.84 [-3.42, 1.74]
Davis 2010	-4.68	5.55	45	-1.82	3.79	45	28.3%	-2.86 [-4.82, -0.90]
Rolls 2017	-1.8	4.6	60	-1.3	4.9	62	35.4%	-0.50 [-2.19, 1.19]
**Subtotal (95% CI)**			**181**			**144**	**100.0%**	**-1.23 [-2.42, -0.04]**
Heterogeneity: Tau² = 0.30; Chi² = 3.75, df = 3 (P = 0.29); I² = 20%								
Test for overall effect: Z = 2.03 (P = 0.04)								
**2.9.3 MR diet + support vs. diet only**								
Shikany 2013	0	3.91	60	-0.07	3.12	60	100.0%	0.07 [-1.20, 1.34]
**Subtotal (95% CI)**			**60**			**60**	**100.0%**	**0.07 [-1.20, 1.34]**
Heterogeneity: Not applicable								
Test for overall effect: Z = 0.11 (P = 0.91)								
**2.9.4 MR diet + enhanced support vs. diet +support**								
Rock 2014 (3)	-6.24	5.4	74	-4.28	5.5	38	50.0%	-1.96 [-4.10, 0.18]
Rock 2014 (4)	-3.48	5.5	77	-4.28	5.5	38	50.0%	0.80 [-1.34, 2.94]
**Subtotal (95% CI)**			**151**			**76**	**100.0%**	**-0.58 [-3.28, 2.12]**
Heterogeneity: Tau² = 2.62; Chi² = 3.20, df = 1 (P = 0.07); I² = 69%								
Test for overall effect: Z = 0.42 (P = 0.67)								
**2.9.5 MR diet + support vs. minimal control**								
Kempf 2018	-2.2	3.6	149	-1.5	3.9	50	6.2%	-0.70 [-1.93, 0.53]
Kempf 2018	-2.8	3.8	160	-1.5	3.9	50	6.1%	-1.30 [-2.53, -0.07]
LookAhead Research Group 2007	-2.85	4.76	2570	-1.72	4.72	2575	72.4%	-1.13 [-1.39, -0.87]
Xu 2013	-3.7	2	46	-1.9	1.6	42	15.3%	-1.80 [-2.55, -1.05]
**Subtotal (95% CI)**			**2925**			**2717**	**100.0%**	**-1.22 [-1.53, -0.90]**
Heterogeneity: Tau² = 0.02; Chi² = 3.37, df = 3 (P = 0.34); I² = 11%								
Test for overall effect: Z = 7.64 (P < 0.00001)								

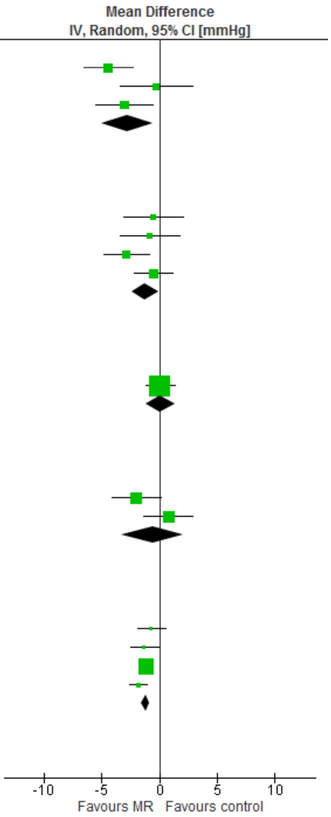

Test for subgroup differences: Chi² = 6.08, df = 4 (P = 0.19), I² = 34.2%

Footnotes
(1) dietician led intervention
(2) physician/nurse led intervention
(3) low fat MR
(4) low carbohydrate MR
